# Evaluation of Anti-Hyperalgesic and Analgesic Effects of Two Benzodiazepines in Human Experimental Pain: A Randomized Placebo-Controlled Study

**DOI:** 10.1371/journal.pone.0043896

**Published:** 2013-03-15

**Authors:** Pascal H. Vuilleumier, Marie Besson, Jules Desmeules, Lars Arendt-Nielsen, Michele Curatolo

**Affiliations:** 1 University Department of Anesthesiology and Pain Therapy, Bern University Hospital, Inselspital, Bern, Switzerland; 2 Division of Clinical Pharmacology and Toxicology, Multidisciplinary Pain Center, University Hospital, Geneva, Switzerland; 3 Center for Sensory–Motor Interaction, Department of Health Science and Technology, Aalborg University, Aalborg, Denmark; California Pacific Medicial Center Research Institute, United States of America

## Abstract

**Background and Aims:**

Compounds that act on GABA-receptors produce anti-hyperalgesia in animal models, but little is known on their effects in humans. The aim of this study was to explore the potential usefulness of GABA-agonism for the control of pain in humans. Two agonists at the benzodiazepine-binding site of GABA_A_-receptors (clobazam and clonazepam) were studied using multiple experimental pain tests. Positive results would support further investigation of GABA agonism for the control of clinical pain.

**Methods:**

In a randomized double-blind crossover design, 16 healthy male volunteers received clobazam 20 mg, clonazepam 1 mg and tolterodine 1 mg (active placebo). The area of static hyperalgesia after intradermal capsaicin injection was the primary endpoint. Secondary endpoints were: area of dynamic hyperalgesia, response to von Frey hair stimulation, pressure pain thresholds, conditioned pain modulation, cutaneous and intramuscular electrical pain thresholds (1, 5 and 20 repeated stimulation), and pain during cuff algometry.

**Results:**

For the primary endpoint, an increase in the area of static hyperalgesia was observed after administration of placebo (p<0.001), but not after clobazam and clonazepam. Results suggestive for an anti-hyperalgesic effect of the benzodiazepines were obtained with all three intramuscular pain models and with cuff algometry. No effect could be detected with the other pain models employed.

**Conclusions:**

Collectively, the results are suggestive for a possible anti-hyperalgesic effect of drugs acting at the GABA_A_-receptors in humans, particularly in models of secondary hyperalgesia and deep pain. The findings are not conclusive, but support further clinical research on pain modulation by GABAergic drugs. Because of the partial results, future research should focus on compounds acting selectively on subunits of the GABA complex, which may allow the achievement of higher receptor occupancy than unselective drugs. Our data also provide information on the most suitable experimental models for future investigation of GABAergic compounds.

**Trial Registration:**

ClinicalTrials.gov NCT01011036

## Introduction

Chronic pain is still a largely unresolved problem. Effective pharmacological approaches need to target alterations in the nociceptive system that are responsible for pain and disability. Extensive animal research has demonstrated that pain conditions are associated with plastic changes of the central nervous system that alter the processing of the somatosensory and nociceptive input [1]. These changes result in reduced pain thresholds to sensory stimuli, enhanced pain after supra-threshold stimulation and enlargement of the areas of local and referred pain. All these manifestations have been consistently detected in human chronic pain [2] and are likely to be highly relevant in terms of amplification of pain and disability. Thus, pharmacological targeting of neuroplastic changes is one important aim of translational pain research.

A relevant aspect of neuroplastic changes in inflammatory and neuropathic conditions is the reduction in inhibitory glycinergic and GABAergic control of dorsal horn neurons: a reduction in the GABA_A_ -mediated endogenous inhibitory control within the central nervous system leads to exaggerated pain and hyperalgesia [3]. Potentiation of GABA_A_ receptor-mediated synaptic inhibition by benzodiazepines reverses pathologically increased pain sensitivity in animal studies [4], [5]. Subtype-selective compounds targeting the alpha2 and/or alpha3 subunit of the GABA_A_ receptor produce antihyperalgesia in mice and rats without sedation and without tolerance induction [6]. These findings open new perspectives for a more selective targeting of pain pathways with GABAergic drugs.

Benzodiazepines produce anti-hyperalgesia in animal models by acting as agonists at the benzodiazepine-binding site of GABA_A_ receptor [4]. These compounds can therefore be studied to explore the potential usefulness of GABA-agonism in human pain conditions. To date, little is known on the effects of GABA_A_ receptor targeting drugs on nociceptive processes in humans. A review on the efficacy of non-opioid analgesics in human experimental pain models did not include these drugs [7]. We are not aware of comprehensive experimental human studies on the efficacy of drugs acting at GABA-receptors. This information is important to evaluate the potential clinical usefulness of these compounds in pain management and to guide future research.

The aim of the present study was to analyze the effect of two agonists at the benzodiazepine-binding site of GABA_A_ receptors (shortly named as benzodiazepines) on different mechanisms of pain processing, using a multimodal experimental testing procedure in healthy volunteers. Clonazepam was studied because it is probably the most commonly prescribed benzodiazepine in chronic pain management. Clobazam is another benzodiazepine that may cause less sedation than clonazepam [8], [9] and might also have anti-hyperalgesic properties. Clobazam has been therefore selected as test compound, with clonazepam as positive control and tolterodine as active placebo.

The ultimate aim of the study was to explore the potential usefulness of GABA-agonism for the control of clinical pain, providing guide for future clinical research. The results were positive for a subgroup of experimental pain modalities, supporting further investigation of GABA-agonism in clinical pain.

## Methods

The protocol for this trial and supporting CONSORT checklist are available as supporting information; see Checklist S1 and Protocol S1.

### Ethic statement

The study was approved by the cantonal ethics committee (No. 152/09), registered in the Clinical Trials Protocol Registration System (NCT01011036), and performed in accordance with the Declaration of Helsinki. Written informed consent was obtained from all participants.

### Design

This is a randomized double-blind crossover study, using pain assessment methods that explore different nociceptive mechanisms. Clobazam 20 mg, clonazepam 1 mg and tolterodine 1 mg (active placebo) were compared. At the end of each session the benzodiazepine antagonist flumazenil 0.2 mg was administered to evaluate whether the observed effects can be reversed.

### Setting

The experiments were performed at the University Department of Anesthesiology and Pain Therapy, Inselspital Bern, Switzerland. Pharmacokinetic and pharmacogenomic investigations were performed at the Division of Clinical Pharmacology and Toxicology, University Hospital of Geneva.

### Subjects

**Figure 1:**
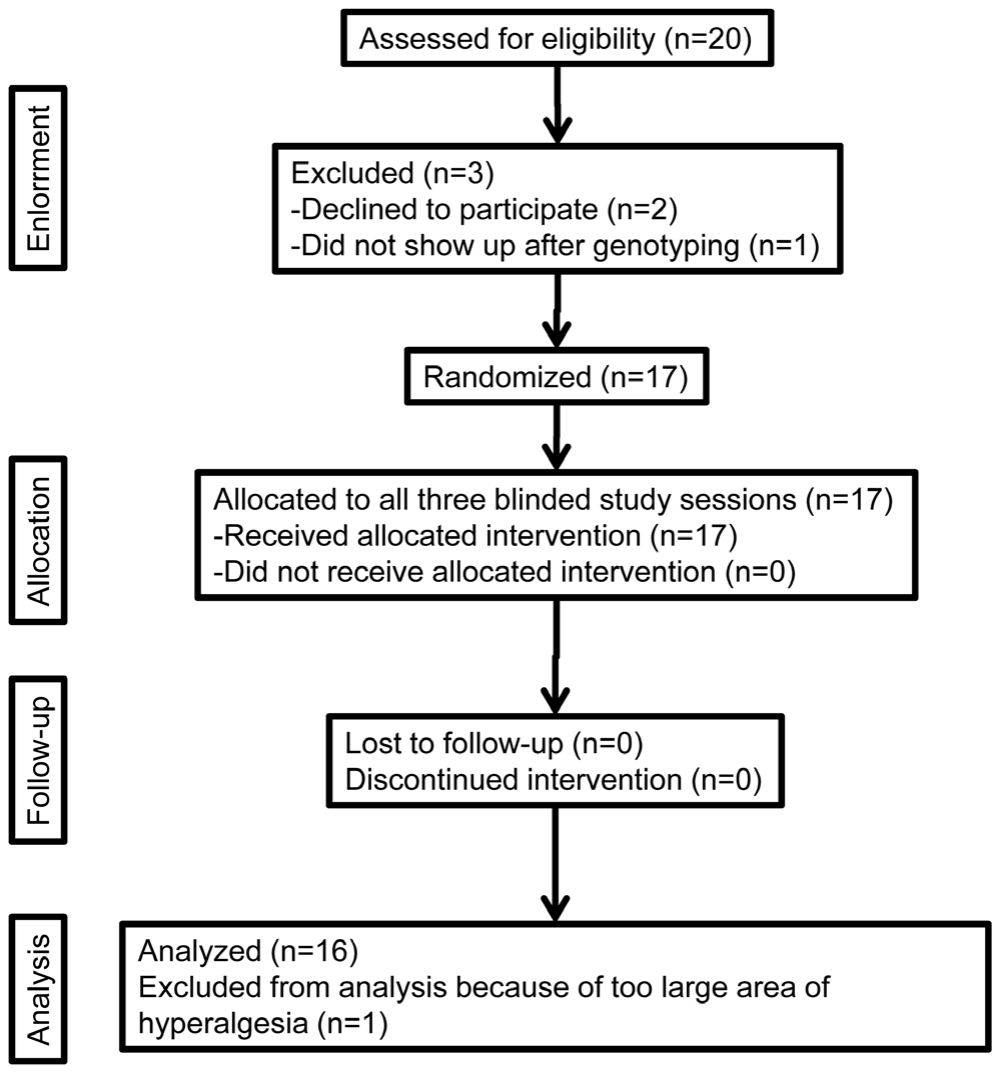
Flowchart of the study.

Volunteers were recruited by advertisement at the Inselspital and at the University of Bern by the first author (P.V.). Sixteen healthy volunteers were tested between December 2009 and July 2010 (figure 1). They received 200 Swiss Francs for each session and a total of 800 Swiss Francs if they completed all the three experiments.

Inclusion criteria were: european ethnicity (in order to minimize pharmacogenetic variations), male gender (in order to avoid possible variation due to hormonal changes during the menstrual cycle), age 18–55 years old, induction of static mechanical hyperalgesia by capsaicin as described below and status as non smoker or moderate smoker (≤10 cigarettes/day).

Exclusion criteria were: current or past history of drug or alcohol abuse, intake of any psychotropic drug currently or in the last month, chronic alcohol intake, any concomitant illness, current or regular intake of any drugs that might affect pain or nociception.

### Study medication

Clobazam, clonazepam and tolterodine were given orally to the same volunteers in a randomized order on three different sessions, with a minimal washout interval between two consecutive sessions of two weeks. All subjects were tested in the morning and had been instructed to take a very light breakfast at home. The minimum interval between breakfast and drug intake was 3 h.

Clobazam (Urbanyl®, Sanofi-Aventis AG, Meyrin, Switzerland) is a benzodiazepine. To our knowledge, it has never been tested for analgesic or antihyperalgesic properties. Unlike 2 mg clonazepam, 20 mg clobazam did not affect significantly cognitive and psychomotor functions, possibly because of its unusual 1–5 chemical structure [9]. Clonazepam (Rivotril®, Roche Pharma AG, Reinach, Switzerland) is probably the benzodiazepine most widely used for the treatment of clinical pain, although the evidence behind this practice is weak [10]. It was therefore chosen as positive control.

The doses of clobazam and clonazepam were chosen based on the following considerations. Clonazepam is typically prescribed in chronic neuropathic pain at doses between 0.5 and 1 mg, and 2 mg a day is an average dose for this indication [11]. Typical anticonvulsant starting doses of clonazepam and clobazam are 0.5–2 mg and 10–20 mg, respectively [12]. A dose of 20 mg of clobazam has been shown to be less sedative than 1 mg of clonazepam and should be equipotent [8]. Therefore these doses have been chosen for the present study.

Tolterodine (Detrusitol®, Pharmacia Gmbh, Pfizer Group, Berlin, Germany) is an anticholinergic compound [13]. Anticholinergic compounds usually cause some sedation and dry mouth; to our knowledge, they are devoid of analgesic effects. Because of its sedative properties, tolterodine has been chosen as an active placebo in order to keep double blinding.

Flumazenil (Anexate®, Roche Pharma AG, Reinach, Switzerland) selectively antagonizes or attenuates the effects of benzodiazepines on GABA_A_ receptors [14]. To minimize the risk of seizure, a dose of 0.2 mg iv was chosen.

The subjects received the study medications in a randomized order. A computer-generated random list was prepared by the hospital pharmacy and was known only by the hospital pharmacy. The drugs were enclosed by the hospital pharmacy in the same type of capsule to assure blinding. The pharmacy delivered the blinded capsules to the investigators, numbered according to the defined randomization order. The first author (P.V.) assigned participants to the experimental sessions.

### Tests

#### Endpoints

The primary endpoint was the area of static hyperalgesia induced by intradermal capsaicin injection. The additional measures were secondary endpoints.

#### General methodological aspects

Because of the very limited information on which experimental pain models would be sensitive to the effects of benzodiazepines, we applied a multimodal testing procedure that is expected to explore different aspects of nociceptive processes. The rationale is summarized in table 1. In particular, we applied models of hyperalgesia, tissue-specific pain sensitivity (cutaneous vs. muscular), sensitivity to short and ongoing painful stimulation, temporal summation (increase in pain perception during stimulation of constant intensity), stimulus-response function (dependency of pain rating from different stimulation intensities) and conditioned pain modulation (exploring endogenous pain modulation).

**Table 1:** Experimental pain tests employed.

Mechanisms explored	Rationale	Methods
Secondary hyperalgesia (primary endpoint)	The drugs could reduce the area of hyperalgesia around the site of primary nociception, which is the result of sensitization of central neural structures	Area of hyperalgesia after intradermal capsaicin injection
Tissue-specific pain sensitivity	The drugs could act differently for nociceptive stimulation arising from different tissues (skin vs. muscle)	Response to cutaneous vs. muscular electrical stimulation
Temporal summation	The drugs could attenuate central summation processes induced by repeated nociceptive stimulation (temporal summation)	Repeated electrical stimulation (5 stimuli) of the skin and muscle
Sensitivity to ongoing painful stimulation	The drugs could be effective for tonic continuous painful stimulation	Cuff algometry and cold pressor test
Stimulus-response function	The effect of drugs could depend on the stimulation intensity	Pain rating after von Frey stimulation at different intensities
Endogenous modulation	The drugs could enhance endogenous inhibitory mechanisms of central pain processes	Conditioned pain modulation (CPM) by ice water test and pressure algometry

Before starting each session, trainings of the pain tests were performed until the subjects were familiar with the testing procedures.

All the tests were applied on the dominant side, except the cold pressor test and cuff algometry (see below for explanations). All subjects but one were right-handed. Each subject was tested on the same side in all three experimental sessions.

Volunteers were not allowed to see the area tested and any read-outs from any instruments.

#### Intradermal capsaicin

Intradermal injection of capsaicin causes a brief stinging/burning pain at the injection site followed by development of secondary hyperalgesia, i.e. hyperalgesia detected at surrounding (not injected) skin [15], [16]. Secondary hyperalgesia after intradermal capsaicin is the result of sensitization of the central nervous system, which is one of the most relevant aspects of neuroplastic changes [17]. This model was sensitive to the action of GABAergic compounds in animal studies [18].

The capsaicin solution was prepared by the Hospital Pharmacy and underwent sterile filtration into sterile septum-sealed vials. The sterile solution (1 mg/ml) was placed into sterile syringes and used for intradermal injection at room temperature. The skin temperature was measured using a digital thermometer, and was 32°C+/−1 in all subjects.

**Figure 2:**
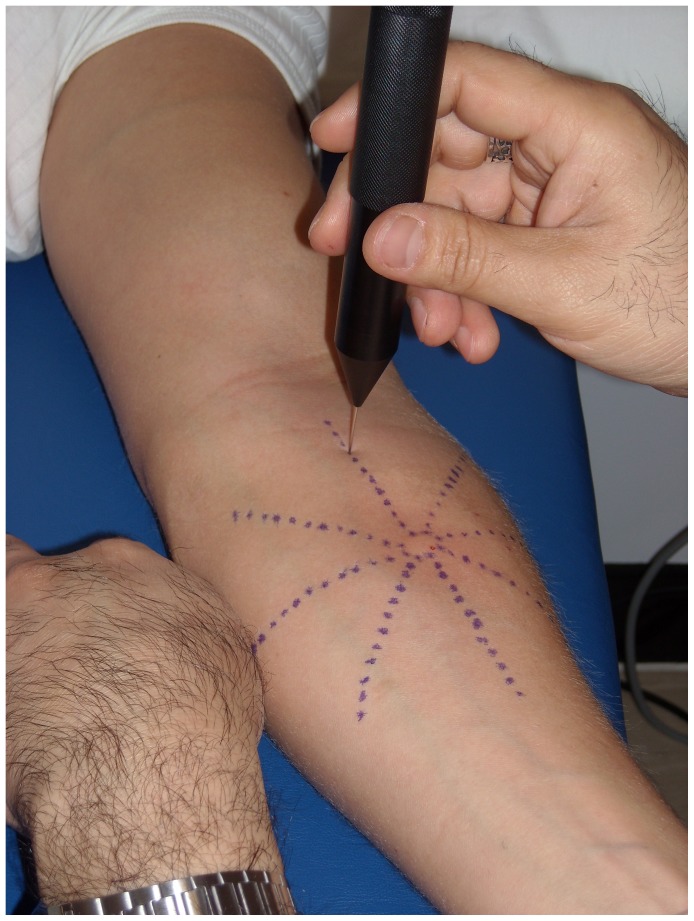
Capsaicin model. The dots represent the points where stimulation was applied. The circle in the center of the hectagon is the site of capsaicin injection.

Subjects remained semi-supine during the experiment. Before capsaicin injection, the skin of the forearm was cleaned using an antiseptic wipe and allowed to air dry. The injection site was marked midway between the elbow and the wrist. Using a surgical skin marker, 4 lines were drawn through the injection site, so that they intersected the corners of a regular octagon (figure 2). These lines were marked every 0.5 cm from the center to the edges using a predefined grid. This resulted in the creation of triangles radiating from the intersection of the 4 lines outwards. We determined the area of hyperalgesia by adding the areas of each triangle in which hyperalgesia was recorded.

Using a 1-ml tuberculin syringe fitted with a 27-gauge disposable needle, 100 µl capsaicin were injected epidermally into the skin, at the intersection of the 4 vectors previously drawn. A white skin elevation appeared during injection ensuring correct injection.

Static mechanical hyperalgesia was judged to be present when the subjects reported that applying a 512 mN von Frey Hair, 5 and 30 minutes after capsaicin injection, elicited pain with an intensity of at least 4 using a numerical rating scale (NRS, whereby 0 = no pain and 10 = worst pain). Only these subjects were included in the study. In order to document pain induction, the subject recorded pain intensity at injection and 5 minutes post-injection using a 10 cm visual analogue pain scale (VAS), whereby 0 = no pain and 10 = worst pain imaginable.

The area of secondary hyperalgesia was assessed using a calibrated 512 mN von Frey hair. The punctuated probe was moved along the 8 radial lines defined above, starting approximately 6 cm away from the site of injection, at each mark in steps of 0.5 cm. The weighted von Frey hair was placed gently on the skin and the load was applied for 2 seconds. The volunteers were asked to report when the pricking sensation changed to a pain sensation. At least 4 seconds elapsed between consecutive stimuli. This procedure was repeated for each vector. The number corresponding to the marker at which sensation changes as described above was noted and the individual numbers were used to calculate the area of hyperalgesia.

The area of dynamic mechanical hyperalgesia was determined by gently stroking a hand-held cotton wool tip on a 1 cm strip of the skin, at a rate of approximately 1 cm/s. Subjects were asked to report when the sensation changed from a non-painful to a painful sensation. The borders of dynamic hyperalgesia were delineated similarly to the determination of static hyperalgesia.

Additionally, mechanical pain sensitivity was assessed using a set of seven weighted pinprick stimuli to obtain a stimulus-response function. The test was applied 1 cm inside the outer border of the pinprick hyperalgesic area, using a set of 7 graded von Frey hair mechanical stimulators with fixed stimulus intensities (custom-made at Aalborg University, Denmark). The flat contact area of the stimulators has a 0.2 mm diameter, and the 7 stimulators exert forces of 8, 16, 32, 64, 128, 256 and 512 mN. The order of stimulation was defined randomly by computer. The stimulators were applied at a rate of 2 s on/2 s off. Subjects were asked to give a pain rating for each stimulus on a 10 cm VAS. Two assessments for each stimulation were made and the mean of these 2 measurements was used for the data analysis.

#### Pressure stimulation

Pain detection and tolerance thresholds were measured with an electronic pressure algometer (Somedic, Hörby, Sweden) applied at the center of the pulp of the 2^nd^ toe. The probe had a surface area of 1 cm^2^. The pressure was increased from 0 at a rate of 30 kPa/s to a maximum pressure of 1200 kPa. Pain detection threshold was defined as the point at which the pressure sensation turned to pain. Pain tolerance threshold was defined as the point at which the subject felt the pain as intolerable. If the threshold was not reached at 1200 kPa, this value was considered as threshold. Three assessments were made and the mean of these 3 measurements were used for the data analysis.

#### Conditioned pain modulation (CPM)

This method explores the endogenous modulation of nociceptive input. Under normal conditions, pain after application of a “test” nociceptive stimulus is attenuated by the application of an additional conditioning noxious stimulus to a remote body region, reflecting diffuse endogenous inhibition [19], [20]. In the present study, pressure pain detection threshold and cold pressure test (see below) were used as test and conditioning stimuli, respectively. An increase in pressure pain detection threshold immediately after cold pressure test was an indication of CPM.

#### Cold pressor test

The subjects placed their hand into a container filled with ice water. In order to maximize heterotopic stimulation, the hand contralateral to the side of pressure stimulation was used. The water was regularly mixed to maintain the temperature near to 0°C. The temperature of the water near the hand was monitored by a thermometer with a digital display (±0.1°C). The subjects were asked to keep the hand in the water until they felt an intolerable sensation of pain and were forced to remove the hand from the container, with a maximum time of 2 min.

#### Pressure stimulation after cold pressor test

Pressure pain detection threshold was measured again at the same time as the subject was withdrawing the hand from the water (one single measurement). CPM was measured as the difference in pressure pain detection threshold between measurements after and before the cold pressure test.

#### Cutaneous single electrical stimulation

Electrical stimulation was performed through electrodes placed distal to the lateral malleolus. A 25 ms, train-of-five, 1 ms, square-wave impulse (perceived as one stimulus), was delivered by a computer-controlled constant current stimulator (Digitimer DS7A, Neurospec, Letchworth Garden City, UK). The current intensity was increased from 1 mA in steps of 0.5 mA until a pain sensation was evoked.

Three assessments were made and the mean of these 3 measurements was used for the data analysis.

#### Cutaneous repeated (5 stimuli) electrical stimulation

The stimulus burst used for single stimulus was repeated 5 times at 2 Hz, at constant intensity. The current intensity of the 5 stimuli was increased from 1 mA in steps of 0.5 mA until the subjects felt pain during the last 2–3 of the 5 stimuli (indicating temporal summation) [21].

Three assessments were made and the mean of these 3 measurements was used for the data analysis.

#### Cutaneous repeated (20 stimuli) electrical stimulation

The stimulus burst used for single stimulus was repeated for a train of 20 pulses at 2 Hz, at an intensity corresponding to the temporal summation threshold. During this 10 s stimulation, pain intensity was continuously rated by the subject with an electronic VAS, and the area under the curve (AUC) was computed. Additionally, the maximal VAS during stimulation was recorded.

#### Intramuscular electrical stimulation (1, 5 and 20 stimuli)

A needle was placed in the tibialis anterior muscle, 14 cm distal from the caudal end of the patella and 20 mm in depth. The same single and repeated stimulation patterns and the same procedure described for cutaneous stimulation were used.

#### Cuff algometry

A tourniquet cuff was applied to the middle of the leg, at the level of the heads of the gastrocnemius and soleus muscle [22]. The cuff was applied at the side contralateral to the side of electrical stimulation in order not to interfere with the positioning of the intramuscular electrodes. During stimulation, the volunteer rated the pain intensity on an electronic VAS scale. The cuff was inflated with compressed air until VAS 6 was reached [22]. The maximum allowed inflating pressure was 200 kPa. The pressure was maintained for 10 min or until the subjects rated the pain as intolerable. The area under the curve VAS-time was computed. For those subjects who felt intolerable pain before 10 min, the time when the cuff was deflated was recorded and VAS 10 was extrapolated until 10 min. Additionally, the maximal VAS during inflation was recorded.

#### Psychomotor performance

The psychomotor performance was assessed by the digit symbol substitution test (DSST), a subscale of the Wechsler Adult Intelligence Scale. The DSST evaluates the ability to concentrate and modifications in processing information [23]. It is a two-minute paper-and-pencil test. The subject was asked to replace digits with corresponding symbols according to a code given on the same sheet of paper. The score consists in the total number and the correct number of symbols drawn.

#### Timing of the experiment

**Figure 3:**
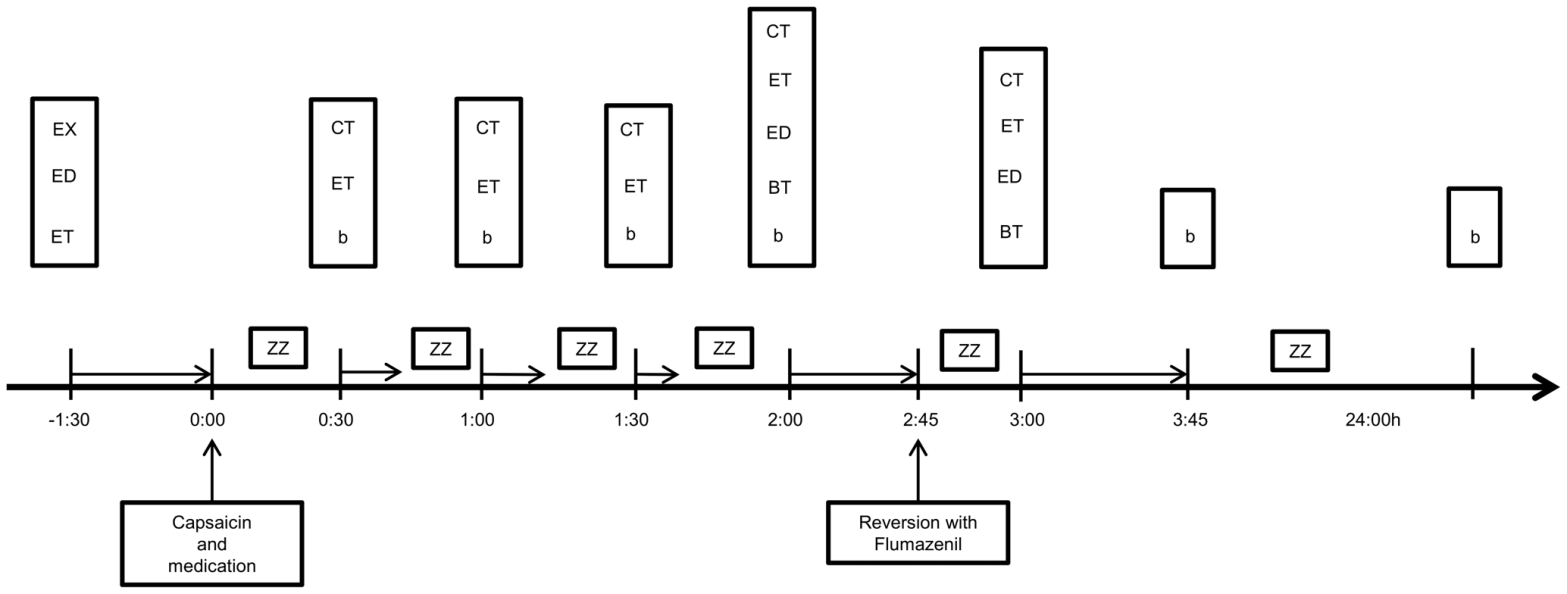
Time plan of the experiment. Horizontal arrow: Testing time; ZZ: Resting time; EX: Medical examination, installation of the testing equipment and training measures; CT: Measures on the area of capsaicin hyperalgesia; ET: Cutaneous electrical stimulation; ED: Intramuscular electrical stimulation, pressure stimulation, cold pressor test, conditioned pain modulation and cuff algometry; BT: Psychomotor performance test; b: Blood sample.

The flow of the experiment is illustrated in figure 3. After training, basal values for the pain tests were determined. The study medication was given immediately after the intradermal injection of capsaicin. Thus, basal values were recorded for all tests, except for those related to capsaicin injection. Because of the relatively short duration of hyperalgesia induced by capsaicin, the recordings of basal values before drug administration would yield a too short testing procedure [17].

The following tests were performed starting 30, 60, 90 and 120 min after medication: all assessments related to capsaicin injection and all cutaneous electrical pain measurements. Pressure pain, conditioned pain modulation (CPM), intramuscular electrical pain tests, cuff algometry and DSST were performed 120 min after drug intake.

Flumazenil was injected 2∶45 h after intake of the study medication. After 15 min, the whole battery of tests was performed.

The occurrence of any side effect was recorded every 30 min, starting at drug administration.

Four hours after intake of the study medication, the volunteers were discharged after control of blood pressure, heart rate and clinical evaluation of cognitive functions.

### Pharmacokinetic and pharmacogenetic investigations

Pharmacokinetic and pharmacogenetic investigations were undertaken on clobazam as the test compound. For pharmacokinetics, blood samples (6 ml) were collected in heparinized tubes at 0.5, 1, 1.5, 2, 4 and 24 hours after drug administration. A specific LC-MS/MS assay was developed and validated in the laboratory of the Division of Clinical Pharmacology and Toxicology of the Geneva University Hospital.

The pharmacogenetic investigation was made in order to control for this factor in case of outlier concentrations of clobazam. It took place 1–4 weeks before the first testing session to evaluate the metabolic activity of cytocrhome P-450 involved in clobazam metabolization.

Phenotype: in vivo activities of 2C19 and 3A4/5 was assessed using micro-doses of omeprazole 2 mg (2C19) and midazolam 0.1 mg (CYP3A4/5), both administered orally at the same time. Two hours after the drugs intake, 2 venous blood samples (6 ml) were collected into heparinized tubes and centrifuged. According to a standardized protocol, the phenotype was determined by calculating the metabolic ratio between deconjugated midazolam and its metabolite 1′-OH-midazolam for CYP3A4 and the ratio of omeprazole and its metabolite OH-omeprazole for CYP2C19 2 h after the drug intake.

Genotype: DNA extracted from blood samples was used for genotype determination. Three single nucleotide polymorphisms (SNP) of the *CYP2C19* gene were determined, CYP2C19*2 (c.681G>A), CYP2C19*3 (c.636G>A) and CYP2C19*17 (g.-806C>T) using polymerase chain reaction-restriction fragment length polymorphism (PCR-RFLP) as described previously.

### Deviations from study protocol

No important changes to methods or outcomes after the trial commenced were done. The following minor deviations from the study protocol were made.

The body side to which the pain tests were applied was not selected by randomization, as originally planned; we decided to perform most tests on the dominant side in order to place the volunteer with the tested side close to the devices; see section 2.6, “general methodological aspects”.

The skin temperature before application of capsaicin was measured, but not recorded; since the skin temperature in the range of 32°C+/−1 was a condition for performing the experiment and no analysis on this parameter was planed, we did not record the individual temperatures; see section 2.6, “intradermal capsaicin”.

For the cold pressor test, we renounced to record the pain intensity continuously using an electronic VAS, because this measurement was not an outcome and in order to simplify the experiment.

The recordings of the DSST test were limited to 120 min and after flumazenil, in order to simplify the experiment.

### Sample size calculation

The primary variable was the area of static hyperalgesia induced by capsaicin injection. The additional measures were secondary variables. No data on the drugs under investigations were available. In a previous study that employed the capsaicin model, pregabalin caused a reduction in the area of hyperalgesia of 10.91 cm^2^, with a standard deviation of 11.54 [24]. This results in a sample size of 11, adopting a 5% level for statistical significance and an 80% power, two-sided. In a conservative prediction of the effects of the drug that we investigated, we planned to study 16 subjects.

### Statistical analysis

The main analysis was a two-way repeated measures ANOVA (SigmaStat 3.5, Systat Software Inc, Chicago, Il, USA), with medication and time as factors of repetition. Non normally distributed data were rank transformed before performing the ANOVA. The analysis tested primarily for the interaction of drug with time (timepoint of the measurements). In case of no significant interaction, we tested the overall effects of the “factors” drug and time. In models with significant interactions, the main effects of the factors drug and time are not interpretable and are therefore not presented. Pairwise comparisons were performed by the Holm-Sidak method.

Because of the high variability of the plasma levels of clobazam (see results), a pharmacokinetic-pharmacodynamic modeling was not feasible. Since the time at which clobazam reached the peak plasma concentration was very variable across subjects, we assumed that its effect might be better detected by considering only the highest drug effect before administration of flumazenil (referred to as “peak value analysis”). We therefore performed a repeated-measure ANOVA by considering as value after medication only the highest drug effect before administration of flumazenil (referred to as “peak value analysis”). This was done for all three drug sessions, in order to keep an unbiased placebo control. For threshold measurements, the peak was the highest value, reflecting the maximum analgesic effect; conversely, for area of hyperalgesia and VAS assessments, the peak analgesic effect corresponded to the lowest value of these parameters.

Finally, we also assumed that including subjects with delayed peak plasma level of clobazam or too low plasma levels during the phase of testing may prevent the detection of drug effects. We therefore repeated the ANOVA analyses after exclusion of subjects with clobazam plasma peaks beyond 2 h after study medication and plasma concentrations lower than 200 µg/ml during the same period (n = 8) (referred to as “subgroup analysis”). The limit of 200 µg/ml was chosen because this is the described mean peak concentration after an oral dose of 10 mg [25].

A p-value<0.05 was considered as statistically significant. Because of the explorative nature of the study, the p-values were not adjusted for multiple comparisons.

## Results

The flowchart of the study is displayed in figure 1. In order to obtain 16 complete experiments, we assessed 20 subjects for eligibility, recruited 18 subjects and randomized 17 of them. In all participants capsaicin elicited pain with an intensity of at least 4 (NRS scale). One of these volunteers was excluded from the analysis because his area of hyperalgesia after capsaicin injection was larger than the vectors drawn on the skin, rendering the measure of the primary endpoint impossible. All the remaining 16 tested male subjects completed the study. Their mean age was 26.1 years (SD 4.2) and the BMI was 22.6 kg/m^2^ (SD 2.6).

The mean values and standard deviations of all performed tests are presented in table 2. For the sake of brevity and to avoid repetition, part of the results of the many statistical analysis are omitted in the following text: here we present the most relevant data, while all statistical analyses are reported in details in table 3. In table 4, an overview of the results is presented.

**Table 2:** Results of the pain tests performed.

Test	Drug	Baseline	30 min	60 min	90 min	120 min	After flumazenil
Area of static hyperalgesia (cm^2^)	TolterodineClonazepamClobazam	n/a	36.3 (10.6)37.9 (13.6)39.7 (18.6)	40.6 (11.5)45.6 (15.3)44.3 (17.1)	43.8 (11.9)43.4 (14.3)43.0 (15.0)	44.7 (10.9)41.9 (11.6)43.6 (16.5)	47.6 (13.7)39.4 (12.3)43.8 (15.0)
Area of dynamic hyperalgesia (cm^2^)	TolterodineClonazepamClobazam	n/a	20.3 (11.6)22.4 (11.2)22.3 (13.2)	23.3 (09.0)19.0 (07.8)22.9 (12.4)	23.1 (11.3)21.7 (12.6)22.4 (13.6)	23.3 (09.1)16.8 (10.8)16.7 (13.0)	24.7 (14.6)19.2 (13.1)21.3 (14.6)
VAS after von Frey hair 8 mN	TolterodineClonazepamClobazam	n/a	1.3 (1.5)1.4 (1.5)1.1 (1.2)	1.2 (1.3)1.2 (0.9)1.9 (3.3)	1.2 (1.4)0.8 (0.8)1.2 (1.4)	1.0 (0.9)1.0 (0.8)1.3 (1.4)	1.1 (1.2)0.6 (0.6)1.1 (1.1)
VAS after von Frey hair 16 mN	TolterodineClonazepamClobazam	n/a	1.7 (1.8)1.6 (1.2)2.0 (2.1)	1.9 (1.9)1.8 (1.9)2.3 (3.2)	1.6 (1.9)1.3 (1.0)1.4 (1.6)	1.4 (1.3)1.4 (1.2)1.3 (1.5)	1.1 (0.9)1.4 (1.2)1.3 (1.1)
VAS after von Frey hair 32 mN	TolterodineClonazepamClobazam	n/a	2.5 (1.9)1.9 (1.5)2.0 (1.6)	2.7 (2.2)2.0 (2.0)2.4 (2.8)	2.2 (1.4)1.8 (1.4)2.1 (2.2)	1.9 (1.3)2.1 (1.7)1.9 (1.9)	2.1 (1.6)1.6 (1.4)1.9 (1.9)
VAS after von Frey hair 64 mN	TolterodineClonazepamClobazam	n/a	3.2 (2.3)2.8 (1.8)2.3 (2.0)	2.9 (1.8)2.7 (2.0)2.5 (2.0)	2.8 (2.0)2.1 (1.6)2.1 (1.9)	2.3 (1.5)2.3 (1.5)2.7 (2.7)	2.6 (1.7)2.2 (1.7)2.2 (1.7)
VAS after von Frey hair 128 mN	TolterodineClonazepamClobazam	n/a	3.4 (2.2)3.2 (4.2)2.9 (2.0)	3.5 (2.2)3.5 (2.0)3.4 (2.7)	3.1 (2.0)2.8 (1.7)3.5 (2.3)	2.9 (2.0)2.9 (1.9)3.0 (2.6)	2.6 (1.5)2.6 (1.9)2.9 (2.1)
VAS after von Frey hair 256 mN	TolterodineClonazepamClobazam	n/a	4.2 (2.1)4.2 (2.2)3.3(2.3)	4.2 (2.1)4.1 (1.8)3.7 (2.6)	4.2 (2.0)4.4 (2.0)3.9 (2.5)	4.1 (2.5)3.9 (2.2)3.8 (2.6)	4.1(1.7)3.8 (1.7)3.9 (2.2)
VAS after von Frey hair 512 mN	TolterodineClonazepamClobazam	n/a	4.9 (2.0)5.2 (2.1)4.5 (2.5)	5.2 (2.0)5.1 (2.0)4.8 (2.8)	4.9 (2.1)5.3 (2.1)4.9 (2.6)	4.8 (2.2)5.0 (1.9)4.7 (2.7)	5.0 (2.2)4.9 (1.7)5.1 (2.5)
Pressure pain detection threshold(kPa)	TolterodineClonazepamClobazam	403 (118)416 (80)432 (123)	n/a	n/a	n/a	371 (88)383 (100)379 (130)	382 (118)389 (112)393 (118)
Pressure pain tolerance threshold(kPa)	TolterodineClonazepamClonazepam	674 (221)685 (172)749 (242)	n/a	n/a	n/a	652 (15)636 (147)712 (262)	685.0 (203)390.8 (199)734.7 (279)
Conditioned pain modulation(kPa)	TolterodineClonazepamClobazam	68 (100)70 (113)71 (98)	n/a	n/a	n/a	72 (114)85 (77)99 (99)	77 (79)74 (86)58 (67)
Pain threshold after singlecutaneous electrical stimulation(mA)	TolterodineClonazepamClobazam	9.9 (2.2)9.7 (3.3)10.0 (2.9)	10.3 (2.6)9.8 (3.5)10.3 (3.6)	10.7 (2.3)10.3 (3.4)10.2 (3.8)	10.9 (2.7)10.6 (3.6)11.2 (3.3)	11.5 (2.3)11.0 (3.6)12.2 (3.9)	12.1 (2.6)11.2 (3.9)12.0 (4.3)
Pain threshold after 5cutaneous electrical stimuli(mA)	TolterodineClonazepamClobazam	8.8 (2.3)8.2 (3.1)8.3 (3.0)	9.0 (2.4)8.2 (3.0)8.7 (3.4)	9.2 (2.2)8.5 (2.9)8.7 (3.4)	9.5 (2.2)8.8 (3.2)9.3 (2.9)	9.7 (2.1)9.1 (3.1)9.9 (3.3)	10.0 (2.3)9.2 (3.3)9.7 (3.3)
Maximal VAS during 20cutaneous electrical stimuli	TolterodineClonazepamClobazam	4.7 (1.5)4.9 (1.6)4.7 (1.8)	5.1 (1.6)5.4 (2.0)4.9 (2.2)	4.8 (1.8)5.0 (2.3)4.7 (1.9)	4.9 (1.8)4.7 (2.1)4.3 (2.0)	4.6 (1.8)4.4 (1.6)4.2 (1.8)	4.7 (1.6)4.2 (1.6)4.5 (1.7)
AUC of VAS during 20cutaneous electrical stimuli	TolterodineClonazepamClobazam	379 (155)359 (144)332 (142)	382 (134)352 (131)338 (152)	362 (155)349 (185)341 (167)	336 (143)351 (141)328 (169)	328 (144)344 (148)314 (146)	326 (136)322 (137)339 (150)
Pain threshold after singleintramuscular electricalstimulation (mA)	TolterodineClonazepamClobazam	1.8 (1.2)1.8 (1.2)1.7 (1.2)	n/a	n/a	n/a	2.2 (1.2)3.0 (2.5)3.1 (4.6)	2.7 (1.7)4.1 (3.7)2.5 (2.1)
Pain threshold after 5intramuscular electricalstimuli (mA)	TolterodineClonazepamClobazam	1.6 (1.2)1.6 (1.1)1.4 (1.1)	n/a	n/a	n/a	1.9 (1.2)2.3 (1.6)2.1 (2.2)	2.2 (1.5)2.9 (2.3)2.2 (2.0)
Maximal VAS during 20intramuscular electricalstimuli	TolterodineClonazepamClobazam	4.5 (1.6)4.3 (1.4)4.2 (1.5)	n/a	n/a	n/a	3.8 (2.4)3.2 (2.4)4.5 (2.5)	4.1 (2.5)2.8 (2.0)4.0 (1.9)
AUC of VAS during 20intramuscular electricalstimuli	TolterodineClonazepamClobazam	323 (127)307 (138)284 (149)	n/a	n/a	n/a	286 (198)236 (204)309 (188)	316 (206)209 (178)292 (169)
AUC of VAS duringcuff algometry	TolterodineClonazepamClobazam	3348 (743)3420 (689)3446 (539)	n/a	n/a	n/a	2686 (1155)3175 (644)3001 (831)	2918 (1021)3225 (671)2932 (566)

The data are expressed as mean (SD). Statistical significance is shown in table 3.

n/a: not applicable. AUC: area under the curve. VAS: visual analog scale for pain (range 0–10).

**Table 3:** Results of the ANOVA analyses on all the tests.

Test	Analysis	Factor Time x Drug	Factor Drug	Factor Time
Area of static hyperalgesia	MainPeakSubgroup	0.016*n/a0.017*	n/p0.781n/p	n/pn/an/p
Area of dynamic hyperalgesia	MainPeakSubgroup	0.291n/a0.131	0.4100.4950.137	0.236n/a0.600
VAS after von Frey hair 8 mN	MainPeakSubgroup	0.686n/a0.482	0.4700.3860.478	0.170n/a0.953
VAS after von Frey hair 16 mN	MainPeakSubgroup	0.925n/a0.796	0.8450.5510.250	0.003*n/a0.053
VAS after von Frey hair 32 mN	MainPeakSubgroup	0.788n/a0.578	0.6720.7350.360	0.169n/a0.210
VAS after von Frey hair 64 mN	MainPeakSubgroup	0.299n/a0.490	0.3260.0790.832	0.094n/a0.388
VAS after von Frey hair 128 mN	MainPeakSubgroup	0.594n/a0.329	0.9370.7980.329	0.009*n/a0.270
VAS after von Frey hair 128 mN	MainPeakSubgroup	0.405n/a0.438	0.4180.7120.326	0.699n/a0.720
VAS after von Frey hair 512 mN	MainPeakSubgroup	0.511n/a<0.001*	0.7720.558n/p	0.329n/an/p
Pressure pain detection threshold	MainPeakSubgroup	0.9270.6060.891	0.7640.7550.609	0.039*0.036*0.134
Pressure pain tolerance threshold	MainPeakSubgroup	0.8810.7500.566	0.1840.1540.194	0.2510.0900.500
Conditioned pain modulation	MainPeakSubgroup	0.8550.8550.725	0.8500.8500.717	0.3460.3460.521
Pain threshold after singlecutaneous electrical stimulation	MainPeakSubgroup	0.2520.3230.087	0.8100.7440.941	<0.001*<0.001*<0.001*
Pain threshold after 5 cutaneouselectrical stimuli	MainPeakSubgroup	0.7700.1690.087	0.6820.7400.819	<0.001*<0.001*<0.001*
Maximal VAS during 20cutaneous electrical stimuli	MainPeakSubgroup	0.3530.1280.593	0.5830.8140.890	<0.001*<0.002*<0.001*
AUC of VAS during 20cutaneous electrical stimuli	MainPeakSubgroup	0.3400.7080.072	0.6280.7020.482	0.1450.063<0.001*
Pain threshold after singleintramuscular electrical stimulation	MainPeakSubgroup	0.1600.4740.109	0.5660.5000.066	0.010*<0.001*0.051
Pain threshold after 5intramuscular electrical stimuli	MainPeakSubgroup	0.1530.3860.042*	0.7790.889n/p	<0.001*0.002*n/p
Maximal VAS during 20intramuscular electrical stimuli	MainPeakSubgroup	0.1050.1320.104	0.2450.0860.047*	0.031*0.026*0.113
AUC of VAS during 20intramuscular electrical stimuli	MainPeakSubgroup	0.1540.2220.589	0.4470.7980.065	0.2110.030*0.311
AUC of VAS during cuff algometry	MainPeakSubgroup	0.3470.2350.161	0.0870.0880.106	0.003*0.002*0.019*
DSST-Score	MainPeakSubgroup	0.095n/a0.040*	0.133n/an/p	<0.001*n/an/p

The table shows the results for the main, the peak value and the subgroup analysis. The main analysis was performed on all subjects for all data. The peak value analysis included only the basal values and the maximal effect before administration of flumazenil. The subgroup analysis included the subjects with peak plasma levels of clobazam before administration of flumazenil and plasma concentrations of at least 200 µg/ml during the same period (n = 8). The table shows the p values for the interaction of drug with time and for the factors drug and time. For analyses with a significant interaction, the results of the effects of the factors drug and time are not interpretable and are therefore not presented (marked as n/p).

* : P-Values <0.05. VAS: pain intensity as assessed by the visual analog scale (0  =  no pain, 10  =  worst pain imaginable). AUC: area under the curve. DSST: digit symbol substitution test (measure of psychomotor performance). n/a: not applicable. n/p: not presented.

**Table 4:** Overview of the results.

Test	Main analysis	Peak value or subgroup analysis	Synthesis of the effects of the GABA-agonists
Area of static hyperalgesia	Significant increase in the area of hyperalgesia only for tolterodine	Decrease in the area of hyperalgesia after Clonazepam	Possible anti-hyperalgesic effect
Area of dynamic hyperalgesia	Not significant	No relevant additional information	No effect
Pain intensity after von Frey stimulation	Not significant	No relevant additional information	No effect
Pressure stimulation	Not significant	No relevant additional information	No effect
Conditioned pain modulation	Not significant	No relevant additional information	No effect
Cutaneous single electrical stimulation	Not significant	No relevant additional information	No effect
Cutaneous repeated (5 stimuli) electrical stimulation	Not significant	No relevant additional information	No effect
Cutaneous repeated (20 stimuli) electrical stimulation	Not significant	No relevant additional information	No effect
Intramuscular single electrical stimulation	Significant increase in pain thresholds only for clonazepam	Significant increase in pain thresholds only for clonazepam in the peak value analysis	Possible analgesic effect
Intramuscular repeated (5 stimuli) electrical stimulation	Significant increase in pain thresholds for clobazam and clonazepam, but not for Tolterodine	Consistent for an increase in pain thresholds after clobazam and clonazepam	Possible analgesic effect
Intramuscular repeated (20 stimuli) electrical stimulation	Not significant	Significantly lower maximal VAS with clonazepam, compared with tolterodine and clobazam in the subgroup analysis	Possible analgesic effect
Cuff algometry	Not significant	Time factor significant, favouring clobazam and clonazepam compared to tolterodine	Possible analgesic effect

The table synthetizes the findings of the the main, peak value and subgroup analysis. The statistical significance for “factors” drug and interaction of drug with time is presented. Significance for “factor” time alone is omitted.

### Area of static hyperalgesia after capsaicin

The pain rating on the VAS-scale immediately after injection of capsaicin was 9.6 (SD 0.7). After five minutes it decreased to 5.8 (SD 1.9). There was no statistically significant difference in the pain rating after among the 3 experimental sessions (p = 0.926 and 0.417 immediately after injection and at 5 min, respectively).

#### Main analysis

**Figure 4:**
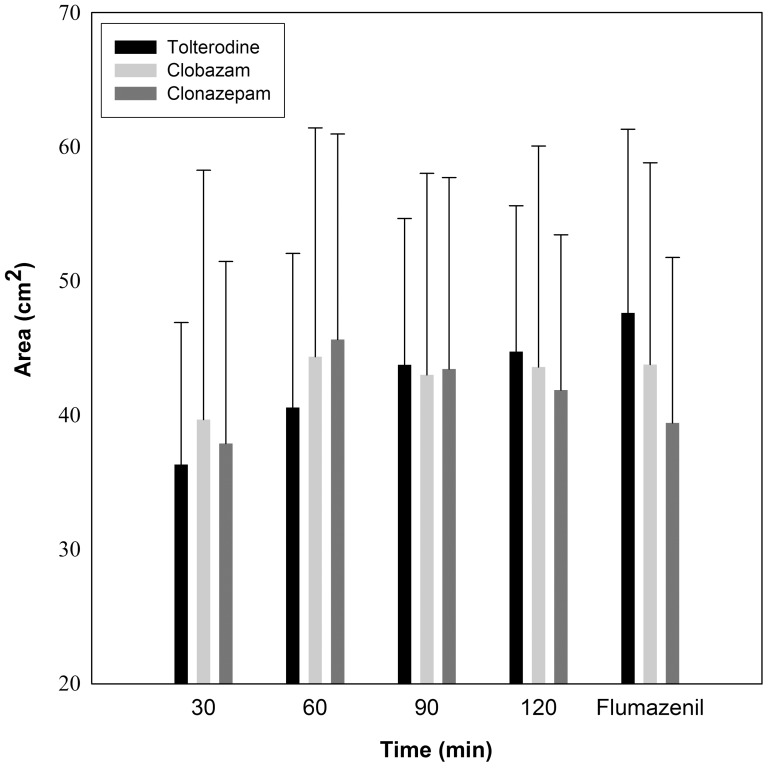
Static pinprick hyperalgesia after capsaicin. The area of static hyperalgesia significantly increased with the active placebo tolterodine (p<0.001), but not with clobazam and clonazepam.

For the area of static hyperalgesia, there was a statistically significant interaction between “factors” drug and time (p = 0.016). Pairwise multiple comparisons within each drug showed that under the placebo tolterodine a significant increase in the area of hyperalgesia over time was observed (p<0.001), whereas there was no statistically significant increase for clonazepam and clobazam (figure 4). No statistically significant difference among the three drug sessions after flumazenil was detected. Thus, capsaicin injection induced a progressive increase in the area of static hyperalgesia, which was prevented by clobazam and clonazepam.

#### Peak value and subgroup analyses

The peak value analysis did not yield statistically significant results. In the subgroup analysis, there was again a significant interaction between time and drug (p = 0.017). Pairwise multiple comparisons within each drug showed that clonazepam significantly decreases the area of hyperalgesia between the time-point 60 min and the measurement after flumazenil (p = 0.004), indicating that flumazenil could not abolish the clonazepam effect.

### Area of dynamic hyperalgesia after capsaicin

No statistical significance was observed.

### Pain intensity after von Frey hair stimulation

In the main analysis, the “factor” drug and interaction between time and drug were not statistically significant for any assessment. The peak value analysis did not yield statistically significant results. In the subgroup analysis, the “factor” interaction of drug with time was significant only for the measurement with 512 mN (p = 0.001). The pairwise multiple comparison within each drug showed an increase of pain rating from the measure at 30 minutes (VAS 3.2, SD 2.8) to the assessment at 120 minutes (VAS 5.1, SD 2.9) with clobazam (p<0.001). This isolated finding is difficult to explain and could be the result of chance.

### Pressure stimulation

For both pain detection and tolerance threshold, the “factor” drug and the interaction between drug and time were not statistically significant for any analysis.

### Conditioned pain modulation (CPM)

No statistical significance was observed.

### Cutaneous single and repeated electrical stimulation (1, 5 and 20 stimuli)

The “factor” drug and the interaction between drug and time were not statistically significant for any analysis.

### Intramuscular single electrical stimulation

#### Main analysis

The “factors” drug and interaction between drug and time were not statistically significant, whereas there was a significant overall time effect (p = 0.01). Pairwise comparisons within each drug revealed a significant increase in the pain threshold only within clonazepam from baseline (1.84, SD1.23) to the time-point after flumazenil (4.05, SD 3.74) (p<0.001).

#### Peak value and subgroup analyses

In the peak value analysis, the “factors” drug and interaction between drug and time were not statistically significant, whereas there was a significant overall time effect (p = 0.015). Pairwise comparisons within each drug revealed clonazepam to significantly increase pain detection thresholds from baseline (9.7, SD 3.3) to peak value (11.0, SD 3.6) (p = 0.032).

The subgroup analysis did not yield statistically significant results.

### Intramuscular repeated (5 stimuli) electrical stimulation

#### Main analysis

**Figure 5:**
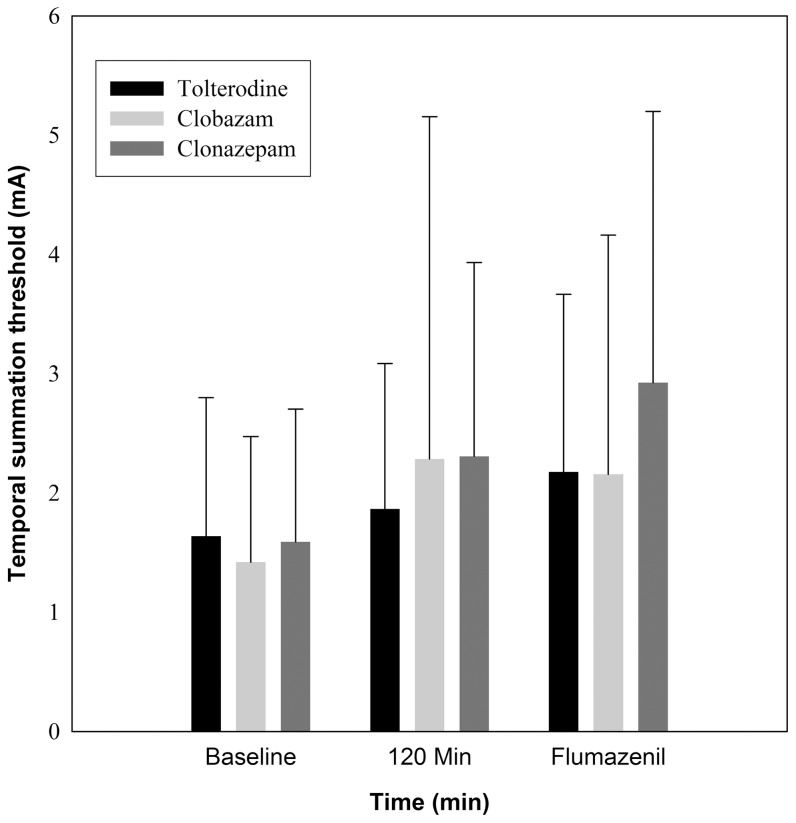
Intramuscular repeated electrical stimulation (5 stimuli). There was a statistically significant increase in the temporal summation thresholds for clonazepam (p = 0.021) and clobazam (p = 0.021) between baseline measures and 120 minutes, whereas no statistically significant change in threshold after the active placebo tolterodine was observed.

The “factors” drug and interaction between drug and time were not statistically significant, whereas there was a significant overall time effect (p<0.001). The pairwise multiple comparison revealed a significant increase in pain thresholds for clonazepam (p = 0.021) and clobazam (p = 0.021) between baseline measures and 120 minutes, whereas no statistically significant change in pain threshold after tolterodine was observed (figure 5).

#### Peak value and subgroup analyses

The peak value analysis confirmed the results of the main analysis. Pairwise comparisons within each drug revealed a significant increase in pain detection threshold from baseline to peak value after clonazepam (p = 0.017) and clobazam (p = 0.007), but not after tolterodine.

In the subgroup analysis, the interaction of drug with time (p = 0.042) was statistically significant. The pairwise multiple comparisons showed that clonazepam significantly increased the pain threshold from baseline (1.69 mA, SD 1.23) to 120 minutes (2.93 mA, SD 2.00) (p<0.001), confirming the above results. The difference persisted also after flumazenil administration (3.58 mA, SD 2.77) (p = 0.004).

### Intramuscular repeated (20 stimuli) electrical stimulation

In the main and peak value analysis, no statistical significance for the “factor” drug and the interaction between drug and time was observed.

In the subgroup analysis the “factor” drug was statistically significant for the maximal VAS (p = 0.047). In the pairwise comparisons among drugs, clonazepam displayed lower values than tolterodine (p = 0.031) and clobazam (p = 0.031).

### Cuff algometry

In the main and peak value analysis, the “factors” drug and the interaction of drug with time were not statistically significant.

In the subgroup analysis, the effect time was significant (p = 0.019), whereas “factors” drug and interaction of drug with time failed to reach statistical significance. In the pairwise multiple comparisons, the AUC after clonazepam was significantly lower than after tolterodine at 120 min (p = 0.024). The AUC after clonazepam significantly decreased from baseline at 120 min (p = 0.003) and after administration of flumazenil (p = 0.007), whereas no significant time effect for the other two drugs was detected.

### Psychomotor performance (DSST-Score)

The mean (SD) of the DSST-score for tolterodine, clobazam und clonazepam 120 min after drug administration was 76.5 (11.7), 74.9 (14.7) and 69.3 (15.8), respectively. After flumazenil, the values for tolterodine, clobazam und clonazepam were 81.5 (10.7), 81.1 (12.1) and 79.1 (14.5), respectively.

In the main analysis, the effect time (p<0.001) was statistically significant whereas the effect drug and the interaction of drug with time failed to show statistical significance. In the pairwise comparison within the time-points, clonazepam (but not clobazam) was significantly associated to a lower score than tolterodine at 120 min (p = 0.031).

In the subgroup analysis, the interaction of drug and time (p = 0.04) was statistically significant. The pairwise multiple comparison showed a lower psychomotor performance with clonazepam at 120 minutes, compared with the other two drugs (p = 0.025). The score within clonazepam significantly increased after flumazenil injection (p = 0.001).

No side effect other than impairment of psychomotor performance was observed.

### Pharmacokinetic and pharmacogenetic investigations

Table 5 presents the pharmacokinetic parameters of clobazam in all subjects (n = 16) and in the subgroup that included the volunteers with peak plasma levels of clobazam before administration of flumazenil (Tmax beyond 180 minutes) and plasma concentrations of at least 200 µg/ml during the same period (n = 8). Five of remaining subjects had a Tmax longer than 180 minutes, which was the time of flumazenil administration. Three more subjects had a maximal concentration (cmax) below 200 µg/ml at 120 minutes. We determined CYP 2C19 phenotype, and one of the 16 subjects was poor CYP2C19 metabolizer.

**Table 5:** Pharmacokinetic parameters of clobazam.

	Tmax (h)	Cmax (ug/ml)	AUC (h*ug/L)	T1/2 (h)
All volunteers (n = 16)	2.28 (1.40)	0.37 (0.17)	5461 (2061)	19.02 (6.55)
Subgroup (n = 8)	1.64 (1.06)	0.47 (0.18)	6160 (1637)	17.50 (2.50)

The table reports the parameters in all volunteers and in the subgroup that included the subjects with peak plasma levels of clobazam below 180 minutes and plasma concentrations of at least 200 µg/ml during the same period (n = 8). Data are expressed as mean values (SD).

## Discussion

This study analyzed the anti-hyperalgesic and analgesic effect of two benzodiazepines in a multimodal experimental pain procedure. The ultimate aim was to explore the potential of GABA-agonism for future clinical research. Interactions between time and drug, results of pairwise comparisons within the ANOVA, and analyses that considered the pharmacokinetic variability are suggestive for a possible anti-hyperalgesic or analgesic effect of GABAergic drugs.

### Primary endpoint

Capsaicin injection induces secondary hyperalgesia, a phenomenon related to central sensitization [26]. The method has been used in several pharmacological studies. It could detect the effects of different drugs, such as opioids, NMDA-antagonists and anticonvulsants [27]–[29], whereas it did not reveal anti-hyperalgesic effects in some studies on cannabinoids and antidepressants [30], [31]. In another study on cannabinoids, the intensity of capsaicin-induced pain, but not the area of hyperalgesia, detected drug effects [32].

We found a statistically significant interaction of the “factors” drug and time. An increase in the area of static hyperalgesia was observed after administration of placebo, but not after clobazam and clonazepam. Our interpretation is that the benzodiazepines prevented the increase in the area of hyperalgesia, thereby displaying an anti-hyperalgesic effect. This is consistent with the large amount of animal data on GABA-agonists [4], [5].

### Secondary endpoints

Results suggestive for an analgesic effect of the benzodiazepines were obtained with all three intramuscular pain models and with cuff algometry. No analgesic effect could be detected with the other pain models employed. The finding that muscle pain models were particularly sensitive to the effects of the benzodiazepines is consistent with a previous investigation on remifentanil: this opioid caused a significantly higher increase in the electrical muscular pain threshold than in the electrical cutaneous pain threshold [33]. The results of these two studies suggest that muscle pain models may be important tools for future pharmacological investigations.

The results with intramuscular stimulation were more robust for the model with repeated (five) stimulations, compared with single stimulation. The 5 repeated stimuli induce temporal summation, a phenomenon related with short-lasting central sensitization [21]. Interestingly, the NMDA-antagonist ketamine increased the pain threshold to repeated stimulation, but had no effect on the pain threshold after single stimulation [21]. This finding is consistent with the established role of NMDA mechanisms in central sensitization [34]. Accordingly, the effects of GABAergic drugs may be better detected with models that induce temporal summation, given the well-know role of GABA-receptors in modulating spinal cord hyperexcitability [3].

### Psychomotor performance

Clonazepam impaired psychomotor performance more than clobazam and tolterodine. This effect was not observed anymore after flumazenil. The results confirm previous data that clobazam may be associated with less sedation than clonazepam, at least at the doses investigated [8], [9].

### Effect of flumazenil

The effect of clobazam and clonazepam was not influenced by flumazenil in most cases. Occasionally, the difference with placebo persisted after administration of this drug. In our opinion, this is likely the result of a too low dose of flumazenil. This dose was chosen in order to minimize the risk of severe complications, such as seizures, in our population of healthy subjects [14].

### Pharmacokinetics and pharmacogenetics

Plasma levels of clobazam exhibited an unexpected high degree of variability. Pharmacogenetic do not explain this variability, since all except one subject were CYP 2C19 extensive metabolizers. The reason for this variability is probably related to delayed absorption. All subjects were tested in the morning and had been instructed to take a very light breakfast at home. Considering the transfer time to the research unit and the additional 2-hours time before drug administration (interview, instruction, training of tests), it is unlikely that our healthy subjects had still some gastric content at the time of drug intake. Delayed absorption, whatever the cause, still remains the most likely explanation for this finding.

### Strengths and limitations

This study implied an extensive exploration of pain mechanisms, including hyperalgesia, cutaneous and muscular pain sensitivity, temporal summation, response to different types of sensory stimuli and conditioned pain modulation. The same investigator performed all the experiments. Pharmacokinetic and pharmacogenetic investigations were included. We are not aware of similar comprehensive studies in this area [7].

The study has also limitations. The capsaicin model does not allow more than 2 h time for testing of hyperalgesia [17]. Based on the pharmacokinetic results, an experimental model with a longer time profile may be more sensitive in detecting the anti-hyperalgesic effects of clobazam or possible active metabolites. Our design implied a single dose administration, and the effect of higher, repeated doses or long-term applications remain unclear.

### Do benzodiazepines display anti-hyperalgesic and analgesic action?

Research on benzodiazepines in clinical pain conditions is sparse. A systematic review on intrathecal midazolam for postoperative pain found some evidence of efficacy [35]. Another systematic review on the efficacy of clonazepam in neuropathic pain and fibromyalgia could not identify any studies that satisfied the inclusion criteria [36].

We detected an effect of the drugs with part of the tests employed. Thus, a conclusive answer to this question cannot be given. However, in the context of experimental work in rodents and humans, this partial result is not surprising. Failure to detect efficacy with part of the experimental pain modalities is a common finding also with drugs characterized by established clinical efficacy [7], [37]. Different experimental pain modalities likely reflect different dimensions of nociceptive processes, as confirmed by a recent large investigation on healthy subjects [38]. Drugs probably affect these dimensions differentially, leading to different sensitivities of pain models to the analgesic effect of drugs.

We cannot rule out that part of the results were positive by chance. However, some arguments are suggestive for a true anti-hyperalgesic and analgesic effect of the drugs investigated. For the primary endpoint, a significant result was obtained with the main analysis on the interaction of drug with time. The result on the primary endpoint is consistent with the findings of basic research. A further argument is the pattern of response that emerges by a collective analysis of the secondary endpoints, as summarized in table 4. None of the pressure pain modalities revealed an effect of clobazam and clonazepam; none of the tests that employed von Frey hair stimulation detected any effect; and none of the cutaneous electrical pain tests yielded any significant result. Conversely, all the three intramuscular pain tests were able to detect analgesic effects of the benzodiazepines. These effects were consistent with the findings obtained with cuff algometry, a model that probably acts, at least in part, by nociceptive stimulation of the muscles. Because of this cluster pattern, it is unlikely that the significant effects that were detected are the result of chance. Rather, they confirm the finding of the aforementioned study, which found responses to different stimulation modalities to represent distinct dimensions of pain perception [38]. Such dimensions seem to be affected differently by analgesic drugs.

Accordingly, our study provides information for future investigations that will analyze the effect of benzodiazepines. The data indicate that models of deep pain and hyperalgesia are probably most suited.

There was no difference between clobazam and clonazepam regarding the effect on the primary endpoint, i.e. the area of static hyperalgesia. However, clonazepam was associated with stronger impairment of psychomotor performance. This suggests that clobazam may offer potential advantages in the clinical management of pain; further clinical investigations on this compound would be desirable.

### Conclusions and perspectives

The present study provides some evidence for an anti-hyperalgesic and analgesic action of agonists at the benzodiazepine-binding site of GABA_A_ receptors. The results are not conclusive, since positive findings were obtained with part of the analyses. Furthermore, the clinical significance of the possible effects of the specific drugs investigated is questionable. Nevertheless, the data support further research in the field of GABA-modulation. GABA-agonists that selectively act at subtypes of GABA_A_-receptors produce potent antihyperalgesia in animal models, in the absence of sedation [6], [39], [40]. Some subtypes of GABA_A_ receptor modulators were investigated in clinical studies for the treatment of anxiety [41], [42]. Analgesic or antihyperalgesic actions of these selective compounds have not been tested in humans. The perspective holds that, due to the minimally sedating properties of these selective drugs, doses higher than for unselective compounds might be used: the consequent higher receptor occupancy could lead to significant anti-hyperalgesic or analgesic effects in clinical pain conditions. Finally, the results of the present study provide information on which models have to be selected for future investigations: methods that induce hyperalgesia and deep pain may be most promising for detecting the effects of GABA_A_-agonists.

## Supporting Information

Checklist S1 **CONSORT Checklist.**
(DOCX)

Protocol S1 **Trial Protocol.**
(PDF)
