# Supplementary material for: Evaluation of Anti-Hyperalgesic and Analgesic Effects of Two Benzodiazepines in Human Experimental Pain: A Randomized Placebo-Controlled Study
Source: PLoS One. 2013 Mar 15;8(3):e43896. doi: 10.1371/journal.pone.0043896 (PMC3598812; doi:10.1371/journal.pone.0043896)
Supplement: Protocol S1 — Trial Protocol. (PDF) [file pone.0043896.s002.pdf]

<u>*Study Protocol*</u>

EFFECTS OF GABA$_A$-AGONISTS ON PAIN MECHANISMS: AN EXPERIMENTAL STUDY IN HEALTHY VOLUNTEERS

Michele Curatolo, M.D., Ph.D.

1st Author: Pascal H. Vuilleumier*

Co-authors: Marie Besson,[#] Jules Desmeules,[#] Alban Y. Neziri,* Hanns U. Zeilhofer,° Lars Arendt-Nielsen,[@]

Senior author: Michele Curatolo*

[#]Division of Clinical Pharmacology and Toxicology, University Hospital of Geneva, Switzerland

*University Department of Anesthesiology and Pain Therapy, Inselspital, Bern, Switzerland.

°Institute of Pharmacology and Toxicology, University of Zurich, Switzerland.

[@]Center for Sensory-Motor Interaction, University of Aalborg, Denmark

18.09.2009

INTRODUCTION

BACKGROUND

Neuropathic and nociceptive pain conditions are associated with plastic changes of the central nervous system [25]. These changes alter the processing of the nociceptive input, thereby leading to reduced pain thresholds to sensory stimuli, enhanced pain after supra-threshold stimulation and enlargement of the areas of local and referred pain [5]. These phenomena have clinical relevance, in that they contribute to pain and disability in patients suffering from chronic pain.

An important aspect of neuroplastic changes in inflammatory and neuropathic conditions is the reduction in inhibitory glycinergic and GABAergic control at dorsal horn neurons [26]. Such dis-inhibitory processes involve reduced responsiveness of certain glycine receptors and changes in the transmembrane chloride gradient of dorsal horn neurons [26]. Potentiation of $GABA_A$ receptor-mediated synaptic inhibition by benzodiazepines reverses pathologically increased pain sensitivity in animal studies [16]. Subtype-selective compounds targeting alpha2 and/or alpha3 subunit containing $GABA_A$ receptors [15] produce antinociception and antihyperalgesia in mice and rats without sedation and without tolerance induction [15]. These findings open new perspectives for a more selective targeting of pain pathways with GABAergic drugs.

Methods that explore specific mechanisms of pain in humans are today available [1]. Such models can be used to assess the effect of drugs on the different mechanisms related to central pain modulation. To date, little is known on the effects of $GABA_A$ receptor targeting

drugs on nociceptive processes in humans. This information is important to evaluate the potential clinical usefulness of these compounds in pain management.

The benzodiazepine that is most commonly prescribed in pain treatment is clonazepam. However, sedation strongly limits its clinical usefulness. Clobazam is another GABAergic compound that may cause less sedation than clonazepam [22; 24]. To our knowledge, its effects on pain processing have not been evaluated.

OBJECTIVE

To evaluate the effect of the $GABA_A$-agonist clobazam on different mechanisms of pain processing by means of a multimodal experimental testing procedure in healthy volunteers. The ultimate aims are twofold. First, we expect information on the potential clinical usefulness of clobazam in the clinical management of pain conditions. Second, compounds acting on subunits of the GABA-receptor complex that may lead to antinociception with no or minimal sedation are becoming available for human research [6]. This may change profoundly the role of GABA-agonists in pain management. The results of the present investigation will provide information on the pain mechanisms that are affected by GABAergic modulation for future investigations on more selective compounds.

METHODS

DESIGN

This is an exploratory, phase II study with a randomized double-blind crossover design, using pain assessment methods that explore different nociceptive mechanisms. In this 3 arms study, clobazam 20 mg will be compared with clonazepam 1 mg (positive control) and tolterodine 1 mg (active placebo). At the end of each session the benzodiazepine antagonist flumazenil 0.2 mg will be administered to evaluate whether the observed effects are GABA-mediated.

SETTING

Department of Anaesthesiology and Pain Therapy, University Hospital, Inselspital Bern, Switzerland. Pharmacokinetic and genetic investigations at the Division of Clinical Pharmacology and Toxicology, University Hospital of Geneva.

SUBJECTS

Sixteen healthy volunteers will be tested after obtaining written informed consent. They will receive 200 Swiss Francs for each session and a total of 800 Swiss Francs if they complete all the three experiments.

INCLUSION CRITERIA

- Caucasian male subjects in order to avoid possible variation due to hormonal changes during the menstrual cycle

- Age: 18 to 55 years old
- Induction of static mechanical hyperalgesia within 5 cm from the injection site of capsaicin
- Non smoker or moderate smoker (≤ 10 cigarettes/day)
- No clinically abnormal findings on history and/or on physical examination

NON-INCLUSION CRITERIA

- Current or past history of drug or alcohol abuse.
- Intake of any psychotropic drug currently or in the last month or chronic alcohol intake
- Any concomitant illness
- Current and regular intake of any drugs that might affect nociception
- If an intercurrent illness necessitating a drug treatment occurs during the assay, a wash-out period of at least 4 half-lives will be left before the next session
- If a volunteer withdraws from the assay ("drop-out") the values obtained hitherto will not be used and another volunteer will be enrolled.

WITHDRAWAL CRITERIA

Volunteers will be informed that they can withdraw at any time without having to give an explanation.

In case of a short-time new diagnosed illness necessitating a drug treatment during the study (upper respiratory tract infection for example), a wash-out period of at least 4 half-lives will be left before the next session.

If needed occasionally during the study, short half-life rescue analgesics (paracetamol, ibuprofen) will be allowed but have to be stopped at least 48 hour before the next experimental session.

In case of withdrawal, another volunteer will be enrolled and the data obtained will not be used in the analysis.

SELECTION AND RECRUITMENT PROCEDURES

Volunteers will be recruited by advertisement at the Inselspital and at the University of Bern. Only Caucasians will be included to minimize the genetic variations in the CYP2C19 gene.

STUDY MEDICATION

CLOBAZAM (URBANYL®)

Pharmacodynamic

Clobazam is a benzodiazepine prescribed in adults and children for all forms of anxiety and as an adjuvant in the treatment of epilepsy. Unlike 2 mg clonazepam, clobazam at the dose of 20 mg did not affect significantly cognitive and psychomotor functions, probably because of its unusual 1-5 chemical structure [22].

Choice of the dosage

The mean recommended dose in “The Swiss Compendium of Drugs” is 0.5/mg/kg daily, which means 35 mg a day for a 70 kg man. Data are on the anti-hyperalgesic properties of clobazam are lacking. The dose of 20 mg has been chosen in comparison with clonazepam. Clonazepam is indeed prescribed in chronic neuropathic pain at doses between 0.5 and 1

mg, and 2 mg a day is an average dose for this indication. Anticonvulsant starting doses of clonazepam and clobazam are 0.5-2 mg and 10-20 mg, respectively. A dose of 20 mg of clobazam has been shown to be less sedative than 1 mg of clonazepam [24]. Therefore these doses have been chosen for the present study.

Safety profile

Like any benzodiazepine, the most common side effects of clobazam are drowsiness, sedation and hangover effect, which are dose-dependent. Sedation occurred in 38% of clobazam-treated patients and 44% of diazepam-treated patients in a placebo-controlled, double-blind trial on 114 outpatients with anxiety[1]. In this study, patients received mean doses of 59 mg clobazam daily and 25 mg diazepam daily. Dizziness occurred in 11% and 2% of diazepam- and clobazam-treated patients. Considering these data, a single dose of 20 mg of clobazam should be well tolerated.

Others common side effects of benzodiazepines such as tolerance and withdrawal syndrome are associated with chronic use and should not be present after a single dose. Finally the risk of respiratory depression is negligible at this dose and in absence of concomitant respiratory pathology [24].

Pharmacokinetics

Clobazam is well absorbed with a 90% biovailability. Maximum plasma concentration is reached within about 2 h (1.3 ± 0.2h after 10 mg and 1.6h ±0.3h after 20 mg in healthy volunteers). Clobazam is mainly metabolized in the liver through the cytochrome P450 enzyme system, notably into N-desmethylclobazam which is an active metabolite [10]. The

---

[1] Micromedex 2009.

half life ($t_{1/2}$) of clobazam is circa 20 h and the $t_{1/2}$ of N-desmethylclobazam is 50 h. These half lifes may be increased in people carrying the *2 variant of the CYP2C19, as it is the main cytochrome implicated in the metabolism of the compound [17].

Considering its high bioavailability, the drug will be administered by mouth. Due to the long $t_{1/2}$ of the parent compound and of the metabolite, each session will take place 2 weeks apart.

Pharmacogenetics

CYP2C19 is polymorphic and people carrying the so-called *2 variant (a single base pair substitution in exon 5 of CYP2C19) have a truncated non functional protein. The activity of CYP2C19 is abolished in homozygous persons and decreased in heterozygous persons [17]. It has been show that the metabolic ratio N-desmethylclobazam/clobazam is higher in people carrying the CYP2C19*2 variant and correlated with more intense adverse effects such as sedation and dizziness [10; 17]. The N-desmethylclobazam/clobazam ratio is also increased after administration of a CYP3A4 inducer such as rifampicine, but to a lesser extend [17].

CLONAZEPAM (RIVOTRIL®)

Pharmacodynamic

Clonazepam is a benzodiazepine prescribed in adults and in children in all forms of epilepsy. Moreover, clonazepam is widely used in practice to treat neuropathic pain and has been demonstrated efficient in myofascial pain [7], temporomadibular joint dysfunction [12], cancer related neuropathic pain [14] and in stomatodynia when used topically [11].

Because of its clinical application in pain medicine, clonazepam is used here as a positive control.

Choice of the dosage.

Doses prescribed in chronic neuropathic pain vary between 0.5 and 2 mg, 1 mg being the average dosage. Based on the starting doses recommended in epilepsy (Swiss Compendium, Documed 2009) clonazepam 1 mg should be as efficient as clobazam 20 mg in terms of anti-hyperalgesic properties.

Safety profile

Clonazepam has the same side effect profile than clobazam with sedation and drowsiness being the most expected side effect. In patients treated for panic disorder, approximately 37% experienced somnolence and 8% experienced dizziness[1].

As for clobazam, others common side effects of benzodiazepines such as tolerance and withdrawal syndrome are associated with chronic use and should not be present after a single dose. Finally the risk of respiratory depression is negligible at this dose and in absence of concomitant respiratory pathology [24].

Pharmacokinetic

Clonazeapm is well absorbed with a 90% biovailability. It is mainly metabolized in the liver trough the cytochrome P450 enzymes system, notably by the CYP3A4. Clonazepam is then excreted by the kidney and the $t_{1/2\ is}$ 20 to 40 hours[2].

---

[1] Micromedex 2009.

[2] Micromedex 2009.

TOLTERODINE (DETRUSITOL®)

Pharmacodynamic

Tolterodine is an anticholinergic compound prescribed in hyperactive bladder. Anticholinergic compounds usually cause some sedation and dry mouth, but are devoid of analgesic effects to the state of our knowledge. Therefore, tolterodine has been chosen as an active placebo.

Choice of the dosage

The starting dose usually recommended in the Swiss Compendium is 2 mg twice a day, which can be decreased to 1 mg twice a day. For tolerance reasons a dose of 1 mg will be given during this study.

Safety profile

The main side effects expected are tachycardia, constipation (6 to 7 %), xerostomia (15 to 30%), dizziness (2%) and somnolence (3%)[1]. These effects are however dose-dependent and should be self-limited after the low single dose chosen.

Pharmacokinetic

Tolterodine is well absorbed with a 77% oral bioavailability. It is metabolised in the liver through CYP2D6, which is polymorphic. In the so-called poor metabolisers, who have a reduced or an abolished activity of CYP2D6, CYP 3A4 is mainly involved in the metabolism of tolterodine. However $t_{1/2}$ of tolterodine is doubled in these people after a single dose. Elimination $t_{1/2}$ is 1.9 to 3.7 hours[2].

---

[1] Micromedex 2009.

[2] Micromedex 2009.

FLUMAZENIL (ANEXATE®)

Pharmacodynamic

Flumazenil selectively antagonizes or attenuates the effects of benzodiazepines on $GABA_A$ receptors by competitively inhibiting their actions at the benzodiazepine binding site of the $GABA_A$ receptor complex. Flumazenil does not alter the pharmacokinetics of benzodiazepines. It is prescribed in benzodiazepine overdose, known or suspected[1].

Choice of the dosage

To avoid a risk of seizure, a dose of 0.2 mg iv will be given.

Safety profile

The main adverse effect of flumazenil is a risk of seizure. However it is essentially seen in patients who are relying on benzodiazepines to control seizures, are physically dependent on benzodiazepines, or who have ingested large doses of other drugs. It should not appear with a single dose of 0.2 mg in benzodiazepines naïve healthy volunteers[2].

Pharmacokinetic

Flumazenil iv is extensively metabolised in the liver with a $t_{1/2}$ of about 50 minutes.

RANDOMIZATION, DRUG PREPARATION AND BLINDING

The subjects will receive the study medications in a randomized order. A computer-generated random list will be prepared by the hospital pharmacy and will be known only by the hospital pharmacy and by an investigator who will not participate in the experiments.

---

[1] Micromedex 2009.

[2] Micromedex 2009.

The drugs will be enclosed by the hospital pharmacy in the same type of capsule to assure blinding. The pharmacy will deliver the blinded capsules to the investigators, numbered according to the defined randomization order.

PAIN TESTS

ENDPOINTS

The mechanisms investigated by the different tests are listed in table 1. The primary endpoint is the area of static hyperalgesia induced by intradermal capsaicin injection. The additional measures will be secondary endpoints.

Table 1. *Effects on mechanisms investigated and methods used.*

**Effects on mechanisms**	**Methods**
Secondary hyperalgesia (primary endpoint)	Area of hyperalgesia after intradermal capsaicin injection
Tissue-specific pain hypersensitivity	Response to cutaneous vs. muscular electrical stimulation
Hypersensitivity to ongoing painful stimulation	Cuff algometry and cold pressor test
Temporal summation	Repeated electrical stimulation (5 stimuli) of the skin and muscle
Stimulus-response function	Pain rating after pressure stimulation at different intensities

Effects on mechanisms	Methods
Endogenous modulation	Diffuse noxious inhibitory control (DNIC) by ice water test and pressure algometry, response to train of 20 electrical stimuli, response to continuous deep tissue stimulation (cuff algometry)

GENERAL METHODOLOGICAL ASPECTS

Before starting the experiment, training sessions of the pain tests will be performed until the subjects will be familiar with the testing procedures.

The body side to which tests are applied and the order of application of the pain tests will be defined before starting the recruitment using a computer-generated randomization. Each subject will be tested on the same side in all three experimental sessions.

Volunteers are not allowed to see the area tested or see any read-outs from any instruments.

INTRADERMAL CAPSAICIN

Several studies have used intradermal capsaicin, a hot tasting ingredient of certain pepper plants, for human experimental pain studies [8; 20]. Intradermal injection of capsaicin causes a brief stinging/burning pain at the injection site followed by development of secondary hyperalgesia, i.e. hyperalgesia detected at surrounding (healthy) skin. Secondary hyperalgesia after intradermal capsaicin is the result of central sensitization [21] and is sensitive to the action of GABAergic compounds in animal studies [16].

Preparation of capsaicin solution

The capsaicin solution will be prepared by the Hospital pharmacy and will undergo sterile filtration into sterile septum-sealed vials. The sterile solution (1 mg/mL) will be placed into sterile syringes and used for intradermal injection at ambient temperature.

Preparation of subjects

Subjects will remain semi-recumbent throughout the experiments and their arm will be held horizontally, supported by a pillow throughout the assessment. Skin temperature will be standardised to 32°C +/- 1°C prior to capsaicin injection and recorded.

Within 2 h before capsaicin injection, the skin of the forearm will be cleaned using an antiseptic wipe and allowed to air dry. The injection site will be marked midway between the elbow and the wrist. Using a surgical skin marker, 4 lines will be drawn through the injection site, so that they intersect the corners of a regular octagon. These lines are marked every 0.5 cm from the center to the edges using a predefined grid and the individual points will be given a number from 1 to 10.

Using a tuberculin syringe (1 ml), fitted with 27-gauge disposable needle, 100 µL capsaicin will be injected epidermally into the skin, at the intersection of the 4 vectors previously drawn. A white wheal should appear during injection ensuring correct injection.

After injection the surrounding skin area becomes hyperalgesic to punctate mechanical stimuli (static hyperalgesia) and to stroking tactile stimulation (dynamic hyperalgesia).

Static mechanical hyperalgesia is judged to be present when a subject indicates that applying a punctuated probe within 5 cm of the injection site elicits a painful perception as compared to the non-painful, pricking perception elicited by the same probe at the contralateral control site. Only these subjects will be included in the study.

Assessment of injection pain

In order to document pain induction, the subject will record pain intensity from injection to 5 minutes post-injection or until the pain intensity returns to zero using a 10 cm electronic visual analogue pain scale (VAS), whereby 0 = no pain and 10 = worst pain imaginable.

Assessment of hyperalgesia

The area of secondary hyperalgesia will be assessed using a calibrated (60 g) von Frey hair. The punctuated probe will be moved along the 8 radial lines defined above, starting approximately 6 cm away from the site of injection, at each mark in steps of 0.5 cm. The weighted von Frey hair is placed gently on the skin and the load is applied for 2 seconds. The volunteers are asked to report when the sensation of the pricking changes to a “different”, “unpleasant” or “burning pain” sensation. More than 4 seconds will elapse between consecutive stimuli in steps of 0.5 cm. This procedure will be repeated for each vector. The number corresponding to the marker at which sensation changes as described above will be noted and the sum of the numbers will be used as a proxy for the area. Additionally, once complete boundaries had been defined, the areas are transcribed onto clear film and weighted.

The area of dynamic mechanical hyperalgesia is determined by gently stroking a hand-held cotton wool tip on the skin at a rate of approximately 1 cm/s. The borders of dynamic hyperalgesia are delineated similarly to the determination of static hyperalgesia. Subjects are asked to report when the sensation changes from a non-painful sensation to a painful/unpleasant sensation by stroking the skin with the cotton wool tip.

Assessment of stimulus-response function

Mechanical pain sensitivity will be assessed using a set of seven weighted pinprick stimuli to obtain a stimulus-response function. The test will be applied 1 cm inside the outer boarder of the pinprick hyperalgesic area, using a set of 7 custom-made graded von Frey hair mechanical stimulators with fixed stimulus intensities. The flat contact area of the stimulators is 0.2 mm diameter, and the 7 stimulators exert forces of 8, 16, 32, 64, 128, 256 and 512 mN. The order of stimulation will be defined randomly by computer. The stimulators will be applied at a rate of 2 s on, 2 s off. Subjects will be asked to give a pain rating for each stimulus on a 10 cm VAS. Two assessments for each stimulation will be made and the mean of these 2 measurements will be used for the data analysis.

PRESSURE PAIN

Pain detection and tolerance thresholds will be measured with an electronic pressure algometer [3] (Algometer, Somedic) applied at the center of the pulp of the 2nd toe. The probe has a surface area of 1 cm^2. The pressure is increased from 0 at a rate of 30 kPa/s to a maximum pressure of 1200 kPa. Pain detection threshold is defined as the point at which the pressure sensation turns to pain. Pain tolerance threshold is defined as the point at which the subject feels pain as intolerable. The subjects are instructed to press a button when these points are reached. The algometer displays the pressure intensity at which the button is pressed. If the subjects do not press the button at a pressure of 1200 kPa, this value is considered as threshold. Three assessments will be made and the mean of these 3 measurements will be used for the data analysis.

For the assessment of the stimulus-response function, stimuli of 1.2, 1.4, 1.6 and 1.8 times the pressure pain detection threshold are applied in randomized order for 2 seconds. After

each stimulation, the volunteer rates the pain intensity on a 10 cm VAS. Two assessments for each stimulation will be made and the mean of these 2 measurements will be used for the data analysis.

DIFFUSE NOXIOUS INHIBITORY CONTROL (DNIC)

This method explores the endogenous modulation of nociceptive input. Under normal conditions, pain after application of a phasic nociceptive stimulus is attenuated by the application of an additional tonic noxious stimulus to a remote body region, reflecting diffuse endogenous inhibition [4; 9]. In the present study, pressure pain detection threshold and cold pressure test are used as phasic and tonic stimuli, respectively. An increase in pressure pain detection threshold immediately after cold pressure test is an indication of DNIC.

Cold pressor test

The device consists of a container separated in an outer and an inner part by a mesh screen. The mesh screen prevents direct contact between the ice (placed in the outer part) and the hand of the subject (placed in the inner part). The outer part of the container is filled with ice that maintains the water on the inner side close to 0°C. The water is regularly mixed to maintain the temperature near to 0°C. The temperature of the water near the hand is monitored by a thermometer with a digital display (±0.1°C).

The subject places his hand, wide open and to the wrist, in the inner part of the container. He is asked to keep it in the water until he feels an intolerable sensation of pain and is forced to remove his hand from the container, with a cut-off time of 2 min.

Pain intensity will be continuously rated by the subject with an electronic VAS. The area under the pain intensity/time curve will be determined. If the hand is withdrawn before the end of the 2 min because of intolerable pain, the pain intensity will be considered to be 10 until the end of the 2 min period.

Pressure pain

Pressure pain detection threshold is measured again at the same time as the subject is withdrawing the hand from the water (one single measurement). DNIC is measured as the difference in pressure pain detection threshold between measurements after and before the cold pressure test.

CUTANEOUS SINGLE ELECTRICAL STIMULATION

Electrical stimulation will be performed through electrodes placed distal to the lateral malleolus. A 25 ms, train-of-five, 1 ms, square-wave impulse (perceived as one stimulus), will be delivered by a computer-controlled constant current stimulator (Digimeter, Neurospec). The current intensity will be increased from 1 mA in steps of 0.5 mA until a pain sensation will be evoked.

Three assessments will be made and the mean of these 3 measurements will be used for the data analysis.

CUTANEOUS REPEATED (5 STIMULI) ELECTRICAL STIMULATION

Temporal summation occurs when repetition of a stimulus increases pain perception. It reflects central integration of nociceptive signals [2]. The stimulus burst used for single stimulus is repeated 5 times at 2 Hz, at constant intensity (Digimeter, Neurospec). The

current intensity of the 5 stimuli is increased from 1 mA in steps of 0.5 mA until the subjects feels pain during the last 2-3 of the 5 bursts (indicating temporal summation). Three assessments will be made and the mean of these 3 measurements will be used for the data analysis.

CUTANEOUS REPEATED (20 STIMULI) ELECTRICAL STIMULATION

The stimulus burst used for single stimulus is repeated for a train of 20 pulses at 2 Hz, at an intensity corresponding to the temporal summation threshold (Digimeter, Neurospec). Previous investigations using reflex assessments indicate that pain intensity may decrease during the stimulation [2], suggesting that this model may investigate endogenous pain modulation. During this 10 s stimulation, pain intensity will be continuously rated by the subject with an electronic VAS.

INTRAMUSCULAR ELECTRICAL STIMULATION (SINGLE AND REPEATED)

A needle is placed in the tibialis anterior muscle, 14 cm distal from the caudal end of the patella and 20 mm in depth. The same single and repeated stimulation patterns described above are used. Three assessments for each stimulation modality will be made and the mean of these 3 measurements will be used for the data analysis.

CUFF ALGOMETRY

To explore response to an ongoing tonic deep-tissue painful stimulus, a tourniquet cuff is applied to the middle of the leg, at the level of the heads of the gastrocnemius and soleus muscle [19], at the side contralateral to the side of electrical stimulation. During the period of stimulation, the volunteer rates the pain intensity on an electronic VAS scale. The cuff will be inflated with compressed air until VAS 6 is reached [19]. The maximum allowed

inflating pressure will be 200 kPa. The pressure will be maintained for 10 min or until the subjects will rate the pain as intolerable. Using this paradigm, part of the subjects experience a reduction in VAS during the stimulation [18], suggesting that the model may explore endogenous pain modulation.

The area under the curve VAS-time is computed. For those subjects who feel intolerable pain before 10 min, the time when the cuff is deflated is recorded and VAS 10 is extrapolated until 10 min for the calculation of the area under the curve.

SIDE EFFECTS

The psychomotor performance will be assessed by the digit symbol substitution test (DSST), a subscale of the Wechsler Adult Intelligence Scale. The DSST evaluates the ability to concentrate and modifications in information processing performance [13]. It is a two-minute paper-and-pencil test. The subject is required to replace digits with corresponding symbols according to a code given on the same sheet of paper. The score consists in the total number and the correct number of symbols drawn. Different versions of the test (i.e., different symbol-digit codes) will be used at each assessment.

The subjective degree of sedation will evaluated with a VAS, whereby 0 = not sleepy at all and 10 = extremely sleepy.

Any other side effect will be recorded.

PHARMACOKINETIC INVESTIGATIONS

Clobazam undergoes extensive metabolism by cytochrome P450 2C19 and CYP3A4.

A specific LC-MS/MS assay will be developed and validated in the laboratory of the Division of Clinical Pharmacology and Toxicology of the Geneva University Hospitals for determination of clobazam and its mean metabolites N-desmethyl clobazam. Blood samples (6 ml) will be collected in heparinized tubes at 0.5, 1, 1.5, 2, 4 and 24 hours after drug administration.

Pharmacokinetic parameters of clobazam can be estimated by an adequate model using WinNonlin version 4.1 (Pharsight Corporation, California, USA).

PK/PD modelling will be performed using WinNonmix (Pharsight Corporation, California, USA) and NONMEM software (San Francisco, California, USA).

PHARMACOGENOMIC INVESTIGATIONS

PHENOTYPING

The investigation will take place once, 1-4 weeks before the first testing session.

In vivo activities of 2C19 and 3A4/5 will be assessed using micro-doses of omeprazole 2 mg (2C19) and midazolam 0.1 mg (CYP3A4/5), both administered orally at the same time. Significant effects (positive or negative) are not expected at such low doses. Two hours after the drugs intake, 2 venous blood samples (6 ml) are collected into heparinized tubes and centrifuged. According to a standardized protocol, the phenotype is determined by calculating the metabolic ratio between deconjugated midazolam and its metabolite 1'-OH-midazolam for CYP3A4 and the ratio of omeprazole and its metabolie OH-omeprazole for CYP2C19 2h after the drug intake.

GENOTYPING

DNA extracted from blood samples is used for genotype determination. Three single nucleotide polymorphisms (SNP) of the *CYP2C19* gene will be determined, *CYP2C19*2* (c.681G>A), *CYP2C19*3* (c.636G>A) and *CYP2C19*17* (g.-806C>T) using polymerase chain reaction-restriction fragment length polymorphism (PCR-RFLP) as described previously.

TIMING OF TESTS

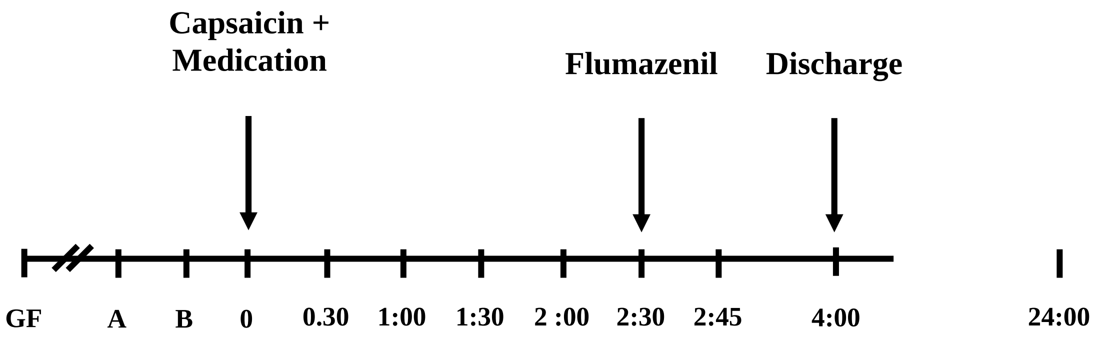

GF: GENOTYPING / PHENOTYPING

This examination takes place once, 1-4 weeks before the first testing session.

- Midazolam-omeprazole test.
- Blood sample for CYP2C19 genotype/phenotype.

A: DEMOGRAPHICS, BLOOD SAMPLE AND TRAINING

- Recordings of age, gender, body height, body weight.
- Training for pain tests: static and dynamic hyperalgesia (without capsaicin injection), pressure pain (detection threshold, tolerance threshold, stimulus-response function),

cold pressor test, cutaneous electrical stimulation (1, 5 and 20 stimuli), intramuscular electrical stimulation (1, 5 and 20 stimuli), cuff algometry.

B: Baseline measurements

- Stimulus-response function by von Frey hairs, pressure pain (detection threshold, tolerance threshold, stimulus-response function), DNIC (cold pressor test with pressure pain detection threshold at the end), cutaneous electrical stimulation (1, 5 and 20 stimuli), intramuscular electrical stimulation (1, 5 and 20 stimuli), cuff algometry.

0

- Administration of study medication.
- Capsaicin injection 5 minutes later
- Assessment of injection pain.

0:30h

- Static and dynamic hyperalgesia, stimulus-response function by von Frey hairs, pressure pain (detection threshold, tolerance threshold, stimulus-response function), cutaneous electrical stimulation (1, 5 and 20 stimuli).
- Blood sample.

1:00h

- Static and dynamic hyperalgesia, stimulus-response function by von Frey hairs, cutaneous electrical stimulation (1, 5 and 20 stimuli).
- DSST.

- Blood sample.

1.30H

- Static and dynamic hyperalgesia, stimulus-response function by von Frey hairs, cutaneous electrical stimulation (1, 5 and 20 stimuli).
- Blood sample.

2:00H

- Static and dynamic hyperalgesia, stimulus-response function by von Frey hairs, pressure pain (detection threshold, tolerance threshold, stimulus-response function), cutaneous electrical stimulation (1, 5 and 20 stimuli), intramuscular electrical stimulation (1, 5 and 20 stimuli), cuff algometry.
- DSST.
- Blood sample.

2:30H

- Administration of flumazenil.

2:45H

15 min after flumazenil administration:

- Static and dynamic hyperalgesia, stimulus-response function by von Frey hairs, pressure pain (detection threshold, tolerance threshold, stimulus-response function), DNIC (cold pressor test with pressure pain detection threshold at the end), cutaneous electrical stimulation (1, 5 and 20 stimuli), intramuscular electrical stimulation (1, 5 and 20 stimuli), cuff algometry.

- DSST.

4:00H (APPROXIMATELY)

- Blood sample.
- Discharge after control of blood pressure, heart rate and clinical evaluation of cognitive functions.

24:00H

- Blood sample.

SAMPLE SIZE CONSIDERATIONS – STATISTICAL ANALYSIS

The primary variable is the area of static hyperalgesia induced by capsaicin injection. The additional measures will be exploratory secondary variables. No data on the drugs under investigations are available for a sample size calculation. In a previous study that employed the capsaicin model, pregabalin caused a reduction in the area of hyperalgesia of 10.91 cm^2, with a standard deviation of 11.54 [23]. This results in a sample size of 11, adopting a 5% level for statistical significance and an 80% power. In a conservative prediction of the effects of the drug that we investigate, we will study 16 subjects.

The data will be analyzed by two-way repeated measures ANOVA.

RELEVANCE

In clinical practice benzodiazepines, and clonazepam in particular, are used as co-analgesics in chronic pain conditions. Their use is based on few randomized trials and mostly on an empirical basis. Sedation, memory impairment and tolerance development strongly limit the clinical usefulness of these compounds in pain therapy. The particular properties of clobazam, which seems to be relatively free of sedative properties, should offer an opportunity to modulate GABAergic pain transmission in humans with a smaller potential for sedation.

There is a need for developing new treatment strategies that modulate the plasticity changes associated with clinical pain conditions. Subtype-selective GABAergic drugs have potential important clinical applications in that they may attenuate such plasticity changes with minimal or no sedation. Such compounds may become available for human pain research in the near future.

No mechanistic study has profiled the action of drugs acting on GABA-A receptors in humans, which significantly impairs our understanding of the full potential of such drugs in pain management. The planned study would provide insights into the efficacy of these drugs on different mechanisms related to pain processing and therefore contribute to clarify their potential role in clinical pain conditions. A positive finding would justify further human studies on the use of new subtype-selective GABAergic drugs that are devoid of sedation and potentially also of other side-effects.

VARIA

GOOD CLINICAL PRACTICE STANDARDS:

The principles established in the GCP guidelines apply to our study.

RISKS

Potential risks of the capsaicin model include temporary burning or stinging sensation at the site of capsaicin injection, irritation, redness, blistering, or severe and persistent burning at the application site. Rarely severe allergic reactions up to anaphylactic shock may occur. An anesthesiologist familiar with reanimation will be present during the experiment.

Possible risk of intramuscular electrical stimulation is local infection.

All other tests are not expected to cause any damage.

General remarks concerning safety:

In case of adverse drug reaction the healthy volunteers will stay and be treated in the University Hospital of Bern as long as needed. Any unexpected adverse drug reaction will be notified to the Swissmedic.

INDEPENDENT DATA MONITORING COMMITTEE

The data quality and control will be reviewed by Dr. Andreas Siegenthaler, MD, Department of Anesthesiology and Pain Therapy, University Hospital of Bern. Controls will be made in intervals of 5 healthy volunteers.

DATA STORAGE

The data will be stored in a locked-up drawer for 5 years in the University Hospital of Bern.

FINANCING AND INSURANCE

This study is sponsored by a grant from the Swiss National Science Foundation (SPUM no. 33CM30_124117)

The public liability of the investigators will be covered by the insurance of the Inselspital Bern.

TIME SCHEDULE

This is a 24 months study, starting in October 2009. Volunteers will enter a 6 weeks double blind, placebo controlled, three arms, randomised period. The last subject will be included in October 2011.

TASKS OF AUTHORS

- Study design: all the authors.
- Draft of the study protocol: Michele Curatolo.
- Instruction of Pascal Vuilleumier in the test procedures: Alban Neziri and Michele Curatolo.
- Logistic organisation: Pascal Vuilleumier and Alban Neziri.
- Performance of the experiments: Pascal Vuilleumier.
- Analysis of data of pain tests: Pascal Vuilleumier, Michele Curatolo and Lars Arendt-Nielsen.

- Pharmacogenomic analyses: Marie Besson, Jules Desmeules.
- Pharmacokinetic analyses: Marie Besson and Jules Desmeules.
- Interpretation of findings: all authors.
- Draft of article: Pascal Vuilleumier, assisted by Michele Curatolo.
- Final version of article: all authors.
- Senior responsibility of all processes: Michele Curatolo.

REFERENCES

[1] Arendt-Nielsen L, Curatolo M, Drewes A. Human experimental pain models in drug development: translational pain research. Curr Opin Investig Drugs 2007;8:41-53.

[2] Arendt-Nielsen L, Sonnenborg FA, Andersen OK. Facilitation of the withdrawal reflex by repeated transcutaneous electrical stimulation: an experimental study on central integration in humans. Eur J Appl Physiol 2000;81:165-173.

[3] Brennum J, Kjeldsen M, Jensen K, Jensen TS. Measurements of human pressure-pain thresholds on fingers and toes. Pain 1989;38:211-217.

[4] Chitour D, Dickenson AH, Le Bars D. Pharmacological evidence for the involvement of serotonergic mechanisms in diffuse noxious inhibitory controls (DNIC). Brain Res 1982;236:329-337.

[5] Curatolo M, Arendt-Nielsen L, Petersen-Felix S. Central hypersensitivity in chronic pain: mechanisms and clinical implications. Phys Med Rehabil Clin N Am 2006;17:287-302.

[6] de Haas SL, de Visser SJ, van der Post JP, de Smet M, Schoemaker RC, Rijnbeek B, Cohen AF, Vega JM, Agrawal NG, Goel TV, Simpson RC, Pearson LK, Li S, Hesney M, Murphy MG, van Gerven JM. Pharmacodynamic and pharmacokinetic effects of TPA023, a GABA(A) alpha(2,3) subtype-selective agonist, compared to lorazepam and placebo in healthy volunteers. J Psychopharmacol 2007;21:374-383.

[7] Fishbain DA, Cutler RB, Rosomoff HL, Rosomoff RS. Clonazepam open clinical treatment trial for myofascial syndrome associated chronic pain. Pain Med 2000;1:332-339.

[8] Gazerani P, Andersen OK, Arendt-Nielsen L. Site-specific, dose-dependent, and sex-related responses to the experimental pain model induced by intradermal injection of capsaicin to the foreheads and forearms of healthy humans. J Orofac Pain 2007;21:289-302.

[9] Ge HY, Madeleine P, Arendt-Nielsen L. Sex differences in temporal characteristics of descending inhibitory control: an evaluation using repeated bilateral experimental induction of muscle pain. Pain 2004;110:72-78.

[10] Giraud C, Tran A, Rey E, Vincent J, Treluyer JM, Pons G. In vitro characterization of clobazam metabolism by recombinant cytochrome P450 enzymes: importance of CYP2C19. Drug Metab Dispos 2004;32:1279-1286.

[11] Gremeau-Richard C, Woda A, Navez ML, Attal N, Bouhassira D, Gagnieu MC, Laluque JF, Picard P, Pionchon P, Tubert S. Topical clonazepam in stomatodynia: a randomised placebo-controlled study. Pain 2004;108:51-57.

[12] Harkins S, Linford J, Cohen J, Kramer T, Cueva L. Administration of clonazepam in the treatment of TMD and associated myofascial pain: a double-blind pilot study. J Craniomandib Disord 1991;5:179-186.

[13] Hindmarch I. Psychomotor function and psychoactive drugs. Br J Clin Pharmacol 1980;10:189-209.

[14] Hugel H, Ellershaw JE, Dickman A. Clonazepam as an adjuvant analgesic in patients with cancer-related neuropathic pain. J Pain Symptom Manage 2003;26:1073-1074.

[15] Knabl J, Witschi R, Hosl K, Reinold H, Zeilhofer UB, Ahmadi S, Brockhaus J, Sergejeva M, Hess A, Brune K, Fritschy JM, Rudolph U, Mohler H, Zeilhofer HU. Reversal of pathological pain through specific spinal GABAA receptor subtypes. Nature 2008;451:330-334.

[16] Knabl J, Zeilhofer UB, Crestani F, Rudolph U, Zeilhofer HU. Genuine antihyperalgesia by systemic diazepam revealed by experiments in GABA-A receptor point-mutated mice. Pain 2009;141:233-238.

[17] Kosaki K, Tamura K, Sato R, Samejima H, Tanigawara Y, Takahashi T. A major influence of CYP2C19 genotype on the steady-state concentration of N-desmethylclobazam. Brain Dev 2004;26:530-534.

[18] Polianskis R, Graven-Nielsen T, Arendt-Nielsen L. Modality-specific facilitation and adaptation to painful tonic stimulation in humans. Eur J Pain 2002;6:475-484.

[19] Polianskis R, Graven-Nielsen T, Arendt-Nielsen L. Spatial and temporal aspects of deep tissue pain assessed by cuff algometry. Pain 2002;100:19-26.

[20] Scanlon GC, Wallace MS, Ispirescu JS, Schulteis G. Intradermal capsaicin causes dose-dependent pain, allodynia, and hyperalgesia in humans. J Investig Med 2006;54:238-244.

[21] Torebjörk HE, Lundberg LE, LaMotte RH. Central changes in processing of mechanoreceptive input in capsaicin-induced secondary hyperalgesia in humans. J Physiol (London) 1992;448:765-780.

[22] van der Meyden CH, Bartel PR, Sommers DK, Blom M, Pretorius LC. Effects of clobazam and clonazepam on saccadic eye movements and other parameters of psychomotor performance. Eur J Clin Pharmacol 1989;37:365-369.

[23] Wang H, Bolognese J, Calder N, Baxendale J, Kehler A, Cummings C, Connell J, Herman G. Effect of morphine and pregabalin compared with diphenhydramine hydrochloride and placebo on hyperalgesia and allodynia induced by intradermal capsaicin in healthy male subjects. J Pain 2008;9:1088-1095.

[24] Wildin JD, Pleuvry BJ, Mawer GE, Onon T, Millington L. Respiratory and sedative effects of clobazam and clonazepam in volunteers. Br J Clin Pharmacol 1990;29:169-177.

[25] Woolf CJ, Salter MW. Neuronal plasticity: increasing the gain in pain. Science 2000;288:1765-1769.

[26] Zeilhofer HU. The glycinergic control of spinal pain processing. Cell Mol Life Sci 2005;62:2027-2035.
